# Dynamical Characteristics of Recurrent Neuronal Networks Are Robust Against Low Synaptic Weight Resolution

**DOI:** 10.3389/fnins.2021.757790

**Published:** 2021-12-24

**Authors:** Stefan Dasbach, Tom Tetzlaff, Markus Diesmann, Johanna Senk

**Affiliations:** ^1^Institute of Neuroscience and Medicine (INM-6) and Institute for Advanced Simulation (IAS-6) and JARA Institute Brain Structure-Function Relationships (INM-10), Jülich Research Centre, Jülich, Germany; ^2^Department of Physics, Faculty 1, RWTH Aachen University, Aachen, Germany; ^3^Department of Psychiatry, Psychotherapy, and Psychosomatics, Medical School, RWTH Aachen University, Aachen, Germany

**Keywords:** neuromorphic computing, spiking neuronal network, network heterogeneity, synaptic-weight discretization, validation, activity statistics

## Abstract

The representation of the natural-density, heterogeneous connectivity of neuronal network models at relevant spatial scales remains a challenge for Computational Neuroscience and Neuromorphic Computing. In particular, the memory demands imposed by the vast number of synapses in brain-scale network simulations constitute a major obstacle. Limiting the number resolution of synaptic weights appears to be a natural strategy to reduce memory and compute load. In this study, we investigate the effects of a limited synaptic-weight resolution on the dynamics of recurrent spiking neuronal networks resembling local cortical circuits and develop strategies for minimizing deviations from the dynamics of networks with high-resolution synaptic weights. We mimic the effect of a limited synaptic weight resolution by replacing normally distributed synaptic weights with weights drawn from a discrete distribution, and compare the resulting statistics characterizing firing rates, spike-train irregularity, and correlation coefficients with the reference solution. We show that a naive discretization of synaptic weights generally leads to a distortion of the spike-train statistics. If the weights are discretized such that the mean and the variance of the total synaptic input currents are preserved, the firing statistics remain unaffected for the types of networks considered in this study. For networks with sufficiently heterogeneous in-degrees, the firing statistics can be preserved even if all synaptic weights are replaced by the mean of the weight distribution. We conclude that even for simple networks with non-plastic neurons and synapses, a discretization of synaptic weights can lead to substantial deviations in the firing statistics unless the discretization is performed with care and guided by a rigorous validation process. For the network model used in this study, the synaptic weights can be replaced by low-resolution weights without affecting its macroscopic dynamical characteristics, thereby saving substantial amounts of memory.

## 1. Introduction

Computational neuronal network models constrained by available biological data constitute a valuable tool for studying brain function. The large number of neurons in the brain, their dense connectivity, and the premise that advanced brain functions involve a complex interplay of different brain regions (Bressler and Menon, 2010) pose high computational demands on model simulations. The human cortex consists of more than 10^10^ neurons (Herculano-Houzel, 2009), each receiving about 10^4^ connections (Abeles, 1991; DeFelipe et al., 2002). The requirements for simulations of networks at this scale by far exceed the limits of modern workstations. Even on high-performance computing (HPC) systems that distribute the work load across many compute nodes running designated simulation software, neuronal networks larger than 10% of the human cortex are not accessible to simulation to date (Jordan et al., 2018). Studying downscaled networks with reduced neuron and synapse numbers does not qualify as an alternative to natural-density full-scale networks: while parameter adjustments can compensate to preserve some characteristics of the network dynamics such as firing rates, or the sensitivity to small perturbations (Bachmann et al., 2020), other features, such as the structure of pairwise correlations in the neuronal activity cannot be maintained simultaneously (van Albada et al., 2015).

The complexity of neuronal network models evaluated on conventional HPC systems is limited by simulation speed and hardware requirements. Routine simulations of large-scale natural-density networks are still not a standard. Even with state-of-the-art software and high-performance machines, simulations of biological processes may take several hundred times longer than the respective processes in the brain (Jordan et al., 2018). Biological processes evolving on long time scales (hours, days, up to years) such as learning and brain development are therefore impossible to simulate in reasonable amounts of time. In addition, the power consumption of large-scale network simulations on HPC systems exceeds the demands of biological brains by orders of magnitude (van Albada et al., 2018). In this study, we address another factor obstructing large-scale neuronal network simulations: the high memory demand (Kunkel et al., 2012, 2014). In simulations performed with the NEural Simulation Tool (NEST) (Gewaltig and Diesmann, 2007), simulation software optimized for this application area, the required memory is mainly used for the storage of synapses (Jordan et al., 2018). While the network model by Jordan et al. (2018) involves dynamic synapses undergoing spike-time dependent plasticity, the problem persists also for the simplest static synapse models characterized by a constant weight and transmission delay. Using double-precision floating point numbers, NEST requires 64 bit of memory for the weight and 24 bit for the delay of each synapse (Kunkel et al., 2014). In the mammalian neocortex, the number of synapses exceeds the number of neurons by a factor of 10^4^. Hence, even small memory demands for individual synapses add up to substantial amounts in brain-scale simulations. Reduced memory consumption leads to faster simulation because the network model can be represented on fewer compute nodes, thereby reducing the time required for communication between nodes. Access patterns of synapses are highly variable due to the random structure of neuronal networks and their sparse and irregular activity. Therefore, also on the individual compute nodes, a reduced memory consumption helps as memory access can better be predicted and more of the required memory fits into the cache.

While these and other limitations may not be overcome using conventional computers built upon the Von Neumann architecture (Backus, 1978; Indiveri and Liu, 2015), the development of novel, brain-inspired hardware architectures promises a solution. Examples for these so-called neuromorphic hardware systems with different levels of maturity are SpiNNaker (Furber et al., 2013), BrainScaleS (Meier, 2015), Loihi (Davies et al., 2018), TrueNorth (Merolla et al., 2014), and Tianjic (Pei et al., 2019). All of these systems are designed after different principles and with different aims (Furber, 2016), and they employ different strategies for handling synaptic weights in an architecture with typically little available memory. SpiNNaker, for instance, saves the weights as 16-bit integer values (Jin et al., 2009) and uses fixed-point arithmetic for the computations. BrainScaleS, instead, utilizes a mixed signal approach where the dynamics of individual neurons are implemented by analog circuits embedded in a silicon wafer, and the weight of each synapse is stored using only 6-bit (Wunderlich et al., 2019). Similarly, GPUs (Knight and Nowotny, 2018; Golosio et al., 2021) and Field Programmable Gate Arrays (FPGAs) (Gupta et al., 2015) are used for simulations of neural networks with reduced numerical precision.

Simulation results obtained with different (neuromorphic) hardware and software systems are hard to compare (Senk et al., 2017; Gutzen et al., 2018; van Albada et al., 2018). A number of inherent structural differences (e.g., numerical solvers) may obscure the role of reduced numerical precision for the network dynamics. Here, we systematically study the effects of a limited synaptic-weight resolution in software-based simulations of recurrently connected spiking neuronal networks. We mimic a limited synaptic-weight resolution by drawing synaptic weights from a discrete distribution with a predefined discretization level. All other parameters and dynamical variables are represented in double precision, and all calculations are carried out using standard double arithmetic in the programming language C++. An exception is the spike times of the neurons which are bound to the time grid spanned by the computation time step *h*. This artificially increases synchronization in the network and introduces a global synchronization error of first order (Hansel et al., 1998; Morrison et al., 2007). The limitation can be overcome by treating spikes in continuous time. This is more costly if only a moderate precision is required but leads to shorter run times of high-precision simulations (Hanuschkin et al., 2010). However, in the models considered here, the errors are dominated by other factors (van Albada et al., 2018). Frameworks like NEST may support both simulation strategies enabling the validation of grid-constrained results by continuous time simulations with minimal changes to the executable model description.

In the field of machine learning, a number of previous studies address the effects of low-resolution weights in artificial neural networks (e.g., Dundar and Rose, 1995; Draghici, 2002; Courbariaux et al., 2014; Gupta et al., 2015; Muller and Indiveri, 2015; Wu et al., 2016; Guo, 2018). For spiking neural networks, competitive performance is reported with weights that can take only binary values {+1, −1} in comparison to models with full-precision weights (e.g., Lu and Sengupta, 2020; Jang et al., 2021; Suarez-Ramirez et al., 2021). These studies, however, do not provide any intuitive or theoretical explanation why a particular weight resolution is sufficient to achieve a desirable network performance. It is therefore unclear to what extent the results of these studies generalize to other tasks or networks. It is particularly difficult to transfer these results to neuroscientific network models: while machine learning networks are typically validated based on the achieved task performance, neuroscience often also focuses on the idle (“resting state”) or task related network activity. In this work, we address the origin of potential deviations in the dynamics of neuronal networks with reduced synaptic-weight resolution from those obtained with a high-resolution “reference” of the same network, and develop strategies to minimize these deviations. For some machine learning algorithms, such as reservoir computing, the two views on performance are related as the functional performance depends on the dynamical characteristics of the underlying neuronal network. In general, however, task performance is not a predictor of network dynamics (and vice versa).

We demonstrate our general approach based on variants of the local cortical microcircuit model by Potjans and Diesmann (2014), the “PD model”. This model represents the cortical natural-density circuitry underneath a 1 mm^2^ patch of early sensory cortex with almost 80, 000 neurons and ~10^4^ synapses per neuron, and explains the cell-type and cortical-layer specific firing statistics observed in nature. To account for the natural heterogeneity in connection strengths, the synaptic weights are normally distributed. The PD model may serve as a building block for brain-size networks because the fundamental characteristics of the cortical circuitry at this spatial scale are similar across different cortical areas and species. In the recent past, the PD model served as a benchmark for several validation studies in the rapidly evolving field of Neuromorphic Computing (Knight and Nowotny, 2018; van Albada et al., 2018; Rhodes et al., 2019; Heittmann et al., 2020; Kurth et al., 2020; Golosio et al., 2021). With this manuscript, we aim to add the aspect of weight discretization to the debate.

The manuscript is organized as follows: section 2 provides details on the discretization methods, the validation procedure, the network model, and the network simulations. The main results of the study are presented in section 3 which, for an overview, can be read without prior reading of section 2. Section 3.1 exposes the pitfalls of a naive discretization of synaptic weights, and section 3.2 proposes an optimal discretization strategy for the given synaptic-weight distribution. For illustration, sections 3.1 and 3.2 are based on a variant of the PD model with fixed in-degrees, i.e., a network where each neuron within a population receives exactly the same number of inputs. In section 3.3, in contrast, the in-degrees are distributed (as in the original PD model), allowing for a generalization of the results. Section 3.4 proposes an analytical approach using mean-field theory to substantiate the simulation results on the role of synaptic-weight and in-degree distributions. Section 3.5 investigates the effect of the simulation duration on the relevance of the employed validation metrics, and the validation performance. The final section 4 summarizes the results and discusses future work toward precise and efficient neuronal network simulations.

## 2. Methods

The general approach of this study is to compare simulations of neuronal networks with differently discretized synaptic weights. To assess whether the weight discretization influences the network dynamics, the statistics of the spiking activity in the networks with discretized weights are compared with the statistics in the reference network with double precision weights. Section 2.1 contains specifications of the neuronal network models employed. The following sections describe the methods used for discretizing the synaptic weights (section 2.2) and for calculating and comparing the network statistics (section 2.3).

### 2.1. Description of Network Models

The present study uses the model of the cortical microcircuit proposed by Potjans and Diesmann (2014), which mimics the local circuit below 1 mm^2^ of the cortical surface, as a reference. Tables 1–4 provide a formal description according to Nordlie et al. (2009). The PD model organizes the neurons into eight recurrently connected populations; an excitatory (E) and an inhibitory (I) one in each of four cortical layers: L2/3E, L2/3I, L4E, L4I, L5E, L5I, L6E, and L6I. The identical current-based leaky integrate-and-fire dynamics with exponentially decaying postsynaptic currents describe the neurons of all populations. Connection probabilities *C*_*YX*_ for connections from population *X* to population *Y* are derived from anatomical and electrophysiological measurements. The weights for the recurrent synapses are drawn from three different normal distributions (*N*_distr_ = 3): mean and standard deviation are $\left({\bar{w}}_{\infty },\Delta w_{\infty }\right)=\left(87.8,8.8\right)\;pA$ for excitatory and $\left({\bar{w}}_{\infty },\Delta w_{\infty }\right)=\left(-351.2,35.1\right)\;pA$ for inhibitory connections. The weights from L4E to L2/3E form an exception as the values are doubled: $\left({\bar{w}}_{\infty },\Delta w_{\infty }\right)=\left(175.6,17.6\right)\;pA$. To account for Dale's principle (Strata and Harvey, 1999), negative (positive) sampled weights of connections that are supposed to be excitatory (inhibitory) are set to zero. The resulting distributions are therefore slightly distorted (for the weight distributions used in this study, this distortion is negligible). Transmission delays are also drawn from normal distributions with different parameters for excitatory and inhibitory connections, respectively. Each neuron receives external input with the statistics of a Poisson point process and a constant weight of 87.8 pA. The simulations are performed with a simulation time step of 0.1 ms and have a duration *T*_sim_ of 15 biological minutes with exceptions in sections 2.3.2 and 3.5. For all simulations, the first second *T*_trans_ = 1 s is discarded from the analysis. The actual observation time is therefore *T*_sim_ − *T*_trans_. For easier readability, all times given in this manuscript always refer to the simulation duration *T*_sim_. The initial membrane potentials of all neurons are randomly drawn from a population-specific normal distribution to reduce startup transients.

**Table 1:** Description of the network model following the guidelines of Nordlie et al. (2009).

**Model summary**	
Structure	Multi-layer excitatory-inhibitory (E-I) network
Populations	8 cortical in 4 layers (L2/3, L4, L5, L6)
Connectivity	Random, independent, population-specific; *fixed in-degree* models and *fixed total number* models
Neuron model	Leaky integrate-and-fire (LIF)
Synapse model	Exponentially shaped postsynaptic currents with normally distributed static weights
Input	Independent fixed-rate Poisson spike trains to all neurons (population-specific in-degree)
Measurements	Spikes

**Table 2:** Description of the network model (continuation of Table 1).

**Connectivity**	
• Connection probabilities *C*_*YX*_ from population *X* to population *Y* with {*X, Y*} ∈ {L2/3, L4, L5, L6} × {E, I}. Values are given in (Potjans and Diesmann, 2014, Table 5). • Self-connections (autapses) are prohibited; multiple connections between neurons (multapses) are allowed.	
*Fixed total number* models	Total number of synapses (Potjans and Diesmann, 2014, Equation 1): (1) $S_{YX}=\frac{\log\left(1-C_{YX}\right)}{\log\left(\left(N_{Y}N_{X}-1\right)/\left(N_{Y}N_{X}\right)\right)}$ In- and out-degrees are binomially distributed.
*Fixed in-degree* models	In-degree: (2) $K_{YX}=\frac{S_{YX}}{N_{Y}}$
**Neuron and synapse model**	
Neuron	Leaky integrate-and-fire neuron (LIF) • Dynamics of membrane potential *V*_*i*_(*t*) for neuron *i*: • Spike emission at times $t_{s}^{i}$ with $V_{i}\left(t_{s}^{i}\right)\geq V_{\theta }$ • Subthreshold dynamics with (3) $$\tau_{\text{m}}=R_{\text{m}}C_{\text{m}}:\tau_{\text{m}}{\dot{V}}_{i}=-V_{i}+R_{\text{m}}I_{i}\left(t\right)\text{ if}\forall s:t\notin \left(t_{s}^{i},t_{s}^{i}+\tau_{\text{ref}}\right]$$ • Reset + refractoriness: $V_{i}\left(t\right)=V_{\text{reset}}\text{if}\forall s:t\in \left(t_{s}^{i},t_{s}^{i}+\tau_{\text{ref}}\right]$ • Exact integration with temporal resolution *h* (Rotter and Diesmann, 1999)
Postsynaptic currents	• Instantaneous onset, exponentially decaying postsynaptic currents • Input current of neuron *i* from presynaptic neuron *j*: (4) $I_{i}\left(t\right)=\sum_{j}J_{ij}\sum_{s}{\text{e}}^{-\left(t-t_{s}^{j}-d_{ij}\right)/\tau_{\text{s}}}\Theta \left(t-t_{s}^{j}-d_{ij}\right)$
Synaptic weights (reference distribution)	• Normally distributed (clipped to preserve sign): (5) $w_{ij}\sim N\left\{{\bar{w}}_{\infty ,YX},\Delta w_{\infty ,YX}^{2}\right\},{\bar{w}}_{\infty ,YX}=g_{YX}\cdot {\bar{w}}_{\infty }$
Spike transmission delays	• Normally distributed (left-clipped at *h*): (6) $d_{ij}\sim N\left\{{\bar{d}}_{X},\Delta d_{X}^{2}\right\}$
Initial membrane potentials	• Normally distributed: (7) $V_{ij}\sim N\left\{{\bar{V}}_{0,X},\Delta V_{0,X}^{2}\right\}$

**Table 3:** Neuron, network, and simulation parameters.

**Neuron and network parameters**										
**Populations and external in-degree**										
**Symbol**		**Value**								**Description**
*X*		L2/3E	L2/3I	L4E	L4I	L5E	L5I	L6E	L6I	Population name
*N*_*X*_		20, 683	5, 834	21, 915	5, 479	4, 850	1, 065	14, 395	2, 948	Size
*K*_*X*, ext_		1, 600	1, 500	2, 100	1, 900	2, 000	1, 900	2, 900	2, 100	External in-degree
**In-degrees in** ***fixed in-degree*** **models**										
*K*_*YX*_		from *X*								
		L2/3E	L2/3I	L4E	L4I	L5E	L5I	L6E	L6I	
	L2/3E	2,200	1,080	980	468	160	0	110	0	
	L2/3I	2,991	861	704	290	381	0	61	0	
	L4E	160	35	1,118	795	33	1	668	0	
to *Y*	L4I	1,481	17	1,814	954	17	0	1,609	0	
	L5E	2,189	375	1,136	32	421	497	297	0	
	L5I	1,166	160	571	13	301	405	125	0	
	L6E	326	39	468	92	286	22	582	753	
	L6I	767	6	75	3	137	9	980	460	
**Total number of synapses in** ***fixed total number*** **models**										
*S*_*YX*_		from *X*								
		L2/3E	L2/3I	L4E	L4I	L5E	L5I	L6E	L6I	
	L2/3E	45,499,804	22,323,576	20,253,647	9,670,918	3,293,577	0	2,271,403	0	
	L2/3I	17,443,694	5,018,762	4,105,338	1,690,073	2,221,212	0	353,460	0	
	L4E	3,503,669	756,561	24,482,849	17,413,575	714,524	7,002	14,624,431	0	
to *Y*	L4I	8,114,253	92,831	9,933,537	5,223,271	87,836	0	8,810,905	0	
	L5E	10,613,575	1,817,058	5,507,804	151,900	2,040,738	2,407,889	1,438,969	0	
	L5I	1,241,436	169,424	607,666	12,851	319,601	430,443	132,414	0	
	L6E	4,681,225	556,108	6,727,569	1,320,233	4,112,224	305,028	837,2649	10,827,677	
	L6I	2,260,836	17,207	220,032	8,078	401,637	25,217	2,888,426	1,354,319	
**Connection parameters and external input**										
**Symbol**			**Value**			**Description**				
${\bar{w}}_{\infty }$			87.81 pA			Reference synaptic strength. All synapse weights are measured in units of ${\bar{w}}_{\infty }$.				
*g*_*YX*_						Relative synaptic strengths:				
			1			*X* ∈ {L2/3E, L4E, L5E, L6E}				
			−4			*X* ∈ {L2/3I, L4I, L5I, L6I}, except for:				
			2			(*X, Y*) = (L4E, L2/3E)				
Δ*w*_∞, *YX*_			$0.1\cdot g_{YX}\cdot {\bar{w}}_{\infty }$			Standard deviation of weight distribution				
${\bar{d}}_{\text{E}}$			1.5 ms			Mean excitatory delay				
${\bar{d}}_{I}$			0.75 ms			Mean inhibitory delay				
Δ*d*_*X*_			$0.5\cdot {\bar{d}}_{X}$			Standard deviation of delay distribution				
ν_ext_			8 s^−1^			Rate of external input with Poisson interspike interval statistics				
*w*_ext_			${\bar{w}}_{\infty }$			Synaptic strength of external input				
**LIF neuron model**										
**Symbol**			**Value**			**Description**				
*C*_m_			250 pF			Membrane capacitance				
τ_m_			10 ms			Membrane time constant				
*E*_L_			−65 mV			Resistive leak reversal potential				
*V*_θ_			−50 mV			Spike detection threshold				
*V*_reset_			−65 mV			Spike reset potential				
τ_ref_			2 ms			Absolute refractory period after spikes				
τ_s_			0.5 ms			Postsynaptic current time constant				

**Table 4:** Neuron, network, and simulation parameters (continuation of Table 3).

**Neuron and network parameters (cont.)**										
**Initial membrane potentials**										
**Symbol**		**Value**								**Description**
*X*		L2/3E	L2/3I	L4E	L4I	L5E	L5I	L6E	L6I	Population name
${\bar{V}}_{0,X}$		−68.28	−63.16	−63.33	−63.45	−63.11	−61.66	−66.72	−61.43	Mean in mV
Δ*V*_0, *X*_		5.36	4.57	4.74	4.94	4.94	4.55	5.46	4.48	Standard deviation in mV
**Simulation parameters**										
**Symbol**					**Value**					**Description**
*T*_sim_					15 min					Simulation duration
*h*					0.1 ms					Temporal resolution
*T*_trans_					1 s					Startup transient

In the original implementation of the model, the total number of synapses between two populations *S*_*YX*_ is derived from an estimate of the total number of synapses in the volume and exactly *S*_*YX*_ synapses are established. Section 3.3 uses this *fixed total number* connectivity. The *fixed in-degree* network models in sections 3.1 and 3.2 determine the in-degrees *K*_*YX*_ by dividing the total number of synapses by the number of neurons in the target population and rounding up to the next larger integer, shown in Equation (2). The rounding ensures that at least one synapse remains for a non-zero connection probability. Table 3 summarizes the resulting values of *S*_*YX*_ and *K*_*YX*_.

### 2.2. Discretization of Synaptic Weights

Computer number formats determine how many binary digits, i.e., bits, of computer memory are occupied by a numerical value and how these bits are interpreted (Goldberg, 1991). Both the number of bits, *N*_bits_, and their interpretation differ for the various floating-point and fixed-point formats deployed in software and hardware. A common format is the IEEE 754 double-precision binary floating-point format (binary64) which allocates 64 bits of memory per value encoding the sign (1 bit), the exponent (11 bits), and the significant precision (52 bits). In general, the upper limit of distinguishable values that a format can represent is $2^{N_{\text{bits}}}$. We here aim to identify a possible lower limit for a bit resolution required to store the synaptic weights in neuronal network simulations without compromising the accuracy of the results. The network models studied in this work assume weights to be sampled from continuous distributions, yielding values in double precision in the respective reference implementations.

To mimic a lower bit resolution, we discretize the distributions and systematically reduce the number of attainable values. On the machine, the values are still represented in double precision, but the degrees of discretization considered are by orders of magnitude coarser than double precision. Our approach is therefore independent of the underlying number format. For generality and explicit distinction from the format-specific *N*_bits_, we define the weight resolution by the number of possible discrete values, *N*_bins_, that a discrete distribution is composed of. In the studied network models, projections between different pairs of neuronal populations are parameterized with weights sampled from *N*_distr_ distributions, for details refer to section 2.1. A weight resolution of *N*_bins_ means that *N*_bins_ weight values are assumed for each of the underlying distributions. The maximum total number of different weights in a network model with discretized weights are therefore *N*_bins_ · *N*_distr_ in addition to potentially different weights not sampled from a distribution, e.g., those used to connect external stimulating devices.

After the reference weight values are sampled from the continuous reference distribution, each one of these sampled weights are subsequently replaced by one of the *N*_bins_ discrete values which are computed according to a discretization procedure as follows: first an interval [*w*_min_, *w*_max_] is defined. Then, the interval is divided into *N*_bins_ bins of equal widths such that the left edge of the first bin is *w*_min_ and the right edge of the last bin is *w*_max_. For each bin, indexed by *i* ∈ [1, …, *N*_bins_], the center value *v*_*i*_ is assumed as the discrete value for that bin:

(8) $$\begin{array}{r} v_{i}=w_{\text{min}}+\left(\frac{1}{2}+i-1\right)\cdot w_{\text{step}}\text{ with }w_{\text{step}}=\frac{w_{\text{max}}-w_{\text{min}}}{N_{\text{bins}}}. \end{array}$$

All weights drawn from the continuous reference distribution falling into a specific bin are replaced by the respective *v*_*i*_, meaning that they are rounded to the nearest discrete value. If a sampled weight coincides with a bin edge, the larger one of the two possible *v*_*i*_ is chosen. However, the probability that a double precision weight drawn from a distribution with a continuous probability density function falls exactly onto the edge of one discrete bin is almost zero. Weights outside of the interval [*w*_min_, *w*_max_] are rounded to the closest discrete values, namely the values of the first or last bin. The two discretization schemes used in this study (“naive” and “moment-preserving”) differ in their choice of the boundaries of the interval.

#### 2.2.1. Naive Discretization of Normal Weight Distribution

Without deeper considerations, it seems reasonable to choose [*w*_min_, *w*_max_] such that the number of originally drawn weights outside of this interval is negligible. As we are studying network models in which the underlying continuous weight distributions are normal distributions with mean ${\bar{w}}_{\infty }$ and standard deviation Δ*w*_∞_, a choice could be as follow:

(9) $$\begin{array}{r} \left[w_{\text{min}},w_{\text{max}}\right]=\left[{\bar{w}}_{\infty }-5\Delta w_{\infty },{\bar{w}}_{\infty }+5\Delta w_{\infty }\right]. \end{array}$$

#### 2.2.2. Moment-Preserving Discretization of Normal Weight Distribution

A better choice for the boundaries of the interval takes the statistical properties of the discrete weights into account. If the reference weights are independently generated according to a probability distribution *p*(*w*), the distribution of the discrete weights is a probability mass function $p^{*}\left(v_{i}\right)=:p_{i}^{*}$ with

(10) $$\begin{array}{l} p_{i}^{*}=\{\begin{array}{lll} F\left(w_{\text{min}}+w_{\text{step}}\right)-F\left(-\infty \right) & \text{if} & i=1\\ F\left(w_{\text{min}}+iw_{\text{step}}\right)-F\left(w_{\text{min}}+\left(i-1\right)w_{\text{step}}\right) & \text{if} & i=2,\\ \;\ldots ,\left(N_{\text{bins}}-1\right)\\ F\left(\infty \right)-F\left(w_{\text{min}}+\left(N_{\text{bins}}-1\right)w_{\text{step}}\right) & \text{if} & i=N_{\text{bins}} \end{array} \end{array}$$

and $F\left(w\right)=\int_{-\infty }^{w}p\left(w^{\prime }\right)\text{d}w^{\prime }$. The statistical properties of the discrete weights are calculated as for any other discrete random variable; mean and standard deviation of the discrete weights are:

(11) $$\begin{array}{r} \;{\bar{w}}_{N_{\text{bins}}}=\overset{N_{\text{bins}}}{\underset{i=1}{\sum }}v_{i}p_{i}^{*}\\ \Delta w_{N_{\text{bins}}}=\sqrt{\overset{N_{\text{bins}}}{\underset{i=1}{\sum }}v_{i}^{2}p_{i}^{*}-{\bar{w}}_{N_{\text{bins}}}^{2}}. \end{array}$$

**Figure 1:**
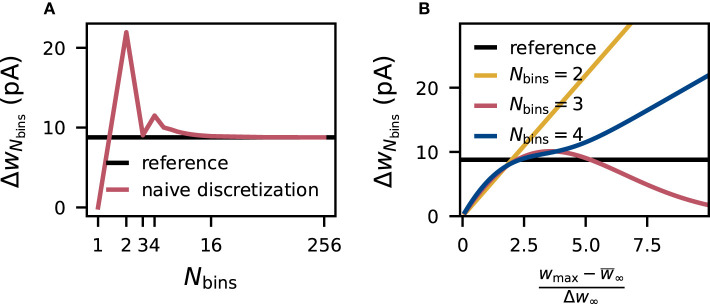
Distortion of synaptic-weight statistics by naive discretization. Dependence of the standard deviation Δ*w*_*N*_bins__ of naively discretized synaptic weights (for excitatory connections) on the number *N*_bins_ of bins **(A)** and on the relative (half-)width $\left(w_{\text{max}}-{\bar{w}}_{\infty }\right)/\Delta w_{\infty }$ of the discretization interval **(B)**. The horizontal black line marks the standard deviation Δ*w*_∞_ of the corresponding (normal) reference weight distribution.

Due to the symmetry of the underlying normal distribution *p*(*w*), the mean of the discrete distribution ${\bar{w}}_{N_{\text{bins}}}$ is always equal to the mean of the continuous reference distribution ${\bar{w}}_{\infty }$ when placing the bins symmetrically around ${\bar{w}}_{\infty }$. On the contrary, the standard deviation Δ*w*_*N*_bins__ of the discrete weights changes with the number *N*_bins_ of bins (Figure 1A). According to Equations (10) and (11), the standard deviation of the discretized version depends on the parameters *w*_min_, *w*_max_ and *N*_bins_. For even numbers of bins, increasing the interval [*w*_min_, *w*_max_] causes the standard deviation to diverge to infinity, and for odd numbers of bins, the standard deviation converges to zero (Figure 1B). In the extreme case of a very wide interval and an odd number of bins, all weights drawn from the normal distribution end up in the central bin and yield identical discrete values causing a standard deviation of zero, while for an even number of bins, the two central bins contain most weights and with growing intervals the discrete values *v*_*i*_ of these two bins drift more and more apart increasing the standard deviation. Therefore, using even numbers of bins the standard deviation of the discrete weights Δ*w*_*N*_bins__ matches the reference standard deviation Δ*w*_∞_ only for one particular choice [*w*_min_, *w*_max_], while using odd numbers of bins leads to a second crossing point. By chance, the naive choice of the interval in Equation (9) is close to the second intersection for three bins. For high numbers of bins, the standard deviation is preserved for a wide range of [*w*_min_, *w*_max_].

The moment-preserving scheme uses the obtained knowledge of the dependence on the standard deviation to improve the discretization procedure: for each number of bins *N*_bins_, different interval boundaries are computed such that the standard deviation is always preserved. This discretization method only preserves the first and second moment, i.e., the mean and standard deviation, respectively, of the underlying reference weight distribution; higher-order moments could still be affected.

For *N*_bins_ = 2 the optimal choice for [*w*_min_, *w*_max_] can be calculated analytically, yielding the interval $\left[{\bar{w}}_{\infty }-2\Delta w_{\infty },{\bar{w}}_{\infty }+2\Delta w_{\infty }\right]$. For any higher number of bins, not only *v*_*i*_ but also $p_{i}^{*}$ in Equation (11) depend on the interval, therefore solutions are found numerically. Here, we use Brent's method implemented in scipy.optimize.root_scalar to find the first intersection. Since the computational effort increases and yields only negligible gain for higher numbers of bins (Figure 1) the optimization is only performed for $N_{\text{bins}}<2^{16}$ and for higher numbers of bins the fixed interval from Equation (9) is used. For *N*_bins_ = 1, this optimization is not possible since the standard deviation is zero by construction.

### 2.3. Validation Procedure

#### 2.3.1. Statistics of Spiking Activity

We evaluate the effects of discretized synaptic weights on network dynamics by employing the same statistical spiking-activity characteristics used in previous studies: distributions of single-neuron firing rates (FR), distributions of coefficients of variation (CV) of the interspike intervals (ISI), and distributions of short-term spike-count correlation coefficients (CC), see Senk et al. (2017), Gutzen et al. (2018), Knight and Nowotny (2018), van Albada et al. (2018), and Golosio et al. (2021). The time-averaged firing rate

(12) $$\begin{array}{r} {\text{FR}}_{i}=\frac{N_{i}\left(T_{\text{trans}},\;T_{\text{sim}}\right)}{T_{\text{sim}}-T_{\text{trans}}} \end{array}$$

of neuron *i* is defined as the total number *N*_*i*_(*T*_trans_, *T*_sim_) of spikes emitted by this particular neuron *i* during the time interval [*T*_trans_, *T*_sim_), normalized by the observation duration (*T*_sim_ − *T*_trans_) (Perkel et al., 1967). The total simulation duration is *T*_sim_, but spike data from the initial interval [0, *T*_trans_) is not analyzed. The ISIs are the time intervals between consecutive spikes of a single neuron. From the ISI distribution, the coefficient of variation

(13) $$\begin{array}{r} {\text{CV}}_{i}=\frac{\sigma_{\text{ISI},i}}{\mu_{\text{ISI},i}} \end{array}$$

of each neuron *i* is computed as the ratio between the ISI standard deviation σ_ISI,*i*_ and its mean μ_ISI,*i*_ (Perkel et al., 1967). The CV is a measure of the spike-train irregularity. In addition to the first-order (single-neuron) measures FR_*i*_ and CV_*i*_, we quantify the level of synchrony in the network on short time scales by the Pearson correlation coefficient

(14) $$\begin{array}{r} {\text{CC}}_{ij}=\frac{C_{ij}\left(0\right)}{\sqrt{C_{ii}\left(0\right)C_{jj}\left(0\right)}} \end{array}$$

for pairs of neurons *i* and *j*. Here,

(15) $$\begin{array}{r} C_{ij}\left(\tau \right)=\langle \left(x_{i}\left(t,t+\Delta \right)-{\langle x_{i}\left(t,t+\Delta \right)\rangle }_{t}\right)\\ \;\times \left(x_{j}\left(t+\tau ,t+\Delta +\tau \right)-{\langle x_{j}\left(t,t+\Delta \right)\rangle }_{t}\right)\rangle_{t} \end{array}$$

denotes the covariance of the spike counts *x*_*i*/*j*_(*t, t* + Δ), i.e., the number of spikes in a time interval [*t, t* + Δ), for a time lag τ (Perkel et al., 1967). The bin size Δ for the covariance calculation matches the refractory period of the neurons in the model networks (2 ms). FR, CV, and CC are calculated using the Python package NetworkUnit (Gutzen et al., 2018) which relies on the package Elephant (Denker et al., 2018).

Note that FR_*i*_ and CC_*ij*_ can only assume discrete values, as the number of spikes *N*_*i*_(*T*_trans_, *T*_sim_) observed in a given time interval of length *D* = *T*_sim_ − *T*_trans_ as well as the spike counts *x*_*i*_ are integer numbers. The discretization of FR_*i*_ with steps ΔFR_*i*_ = 1/*D* usually goes unnoticed if the observation duration *D* is sufficiently large and the FR is not too small. As shown in the following, finite observation times may however affect the shape of the distribution of CC_*ij*_ across pairs of neurons, in particular, if the distribution of FR is narrow (see, e.g., Figures 3F, 4F). With a bin size Δ, the spike counts *x*_*i*_ form vectors of length *M* = *D*/Δ. The scalar product *G*_*ij*_ = *x*_*i*_ · *x*_*j*_ can be regarded as the total spike coincident count for the two neurons *i* and *j* (for small bin sizes Δ and hence binary vectors *x*_*i*/*j*_, it corresponds to the number of bins containing a “1” in both *x*_*i*_ and *x*_*j*_). With the spike-count covariance *C*_*ij*_(0) = *G*_*ij*_/*M* − *N*_*i*_/*M* · *N*_*j*_/*M* and variances $C_{ii/jj}\left(0\right)=N_{i/j}/M-N_{i/j}^{2}/M^{2}$, the CC is given by

(16) $$\begin{array}{r} {\text{CC}}_{ij}=\frac{G_{ij}-N_{i}N_{j}/M}{\sqrt{N_{i}N_{j}\left(1-N_{i}/M\right)\left(1-N_{j}/M\right)}}. \end{array}$$

As the coincidence count *G*_*ij*_ is an integer number, the CC can assume only discrete values with a discretization level

(17) $$\begin{array}{r} \Delta {\text{CC}}_{ij}=\frac{1}{\sqrt{N_{i}N_{j}\left(1-N_{i}/M\right)\left(1-N_{j}/M\right)}}. \end{array}$$

For large *M* (i.e., small bin sizes Δ or long observation durations *D* or both) and small total spike counts *N*_*i*/*j*_ ≪ *M*, the discretization level is given by $\Delta {\text{CC}}_{ij}\approx 1/\sqrt{N_{i}N_{j}}.$ For a heterogeneous population of neurons with different firing rates FR_*i*_ = *N*_*i*_/*D*, the discretization levels ΔCC_*ij*_ are different for each pair of neurons and will hardly affect the distribution of CC. In homogeneous networks where all neurons fire with a similar rate FR≈ FR_*i*_ (∀*i*), however, the distribution of CC may exhibit clear peaks at distances ΔCC = 1/(FR · *D*). For large FR and observation duration, the discretization level ΔCC is small and barely observable. For smaller rates, however, the effect can become striking, even for a relatively long observation duration. With an observation duration *D* = 15min and FR = 1/s, for example, the CCs are discretized with ΔCC ≈ 0.001. In populations 2/3E and 6E in Figures 3F, 4F, this discretization level is only marginally smaller than the population averaged CC, and leads to a pronounced oscillatory pattern in the distributions of CC. Note that the above derivation does not make any assumptions on the higher-order spike train statistics. The discretization level is exclusively determined by the firing rates and the observation duration and is independent of the total coincidence counts. Further, the above arguments are not limited to small bin sizes Δ, but can immediately be generalized.

##### 2.3.1.1. Comparison of Distributions

**Figure 2:**
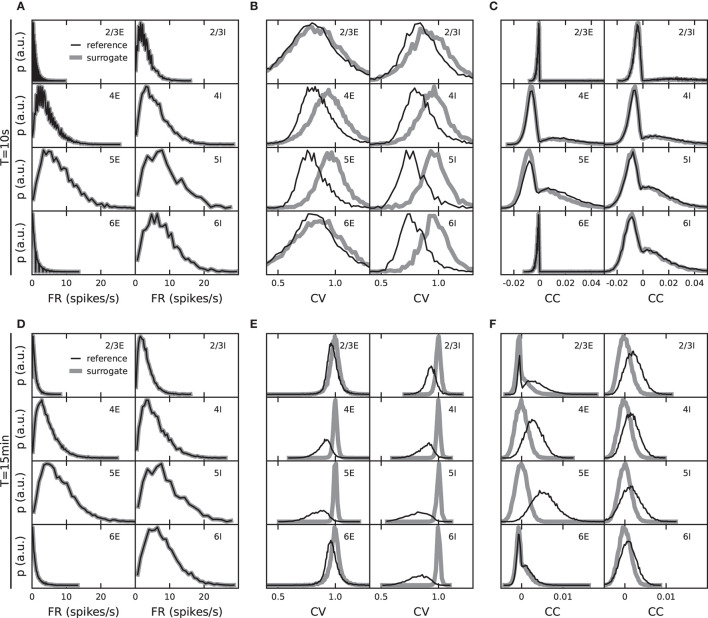
Role of observation duration for the specificity of validation measures. Distributions of population-specific single-neuron firing rates, FR, **(A,D)**, coefficients of variation, CV, of the interspike intervals (ISI) **(B,E)**, and spike-train correlation coefficients, CC **(C,F)**. Black: *fixed total number* network model with reference weight distribution. Gray: surrogate data with randomized spike times (see text). Top: observation duration *T*_sim_ = 10 s. Bottom: *T*_sim_ = 15 min.

**Figure 3:**
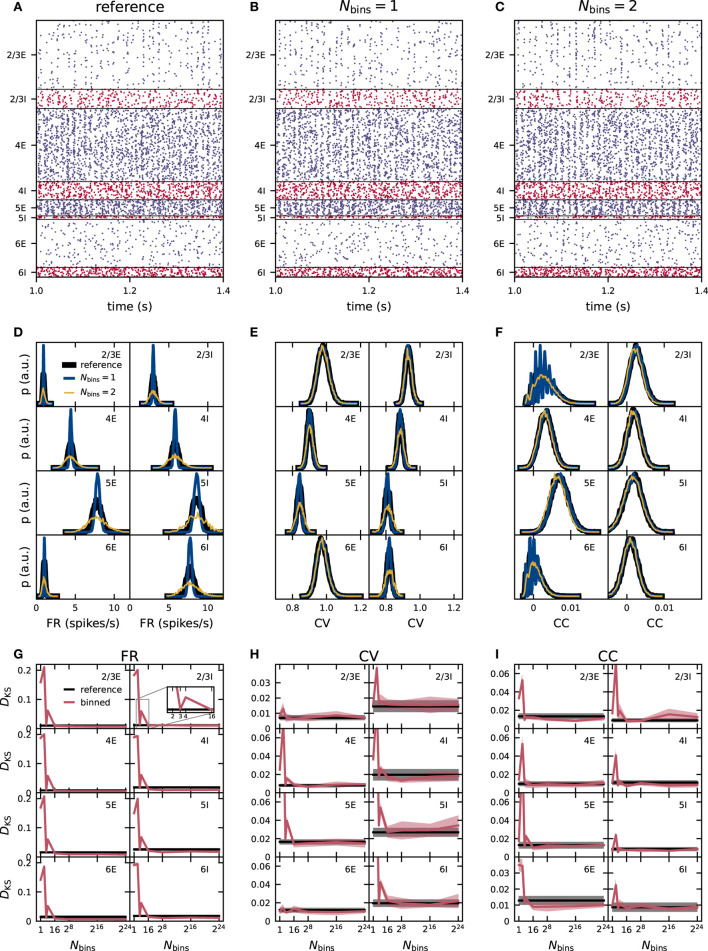
Effect of naive weight discretization on the spike-train statistics in networks with fixed in-degrees. **(A–C)** Spiking activity (dots mark time and sender of each spike) of 5% of all excitatory (blue) and inhibitory (red) neurons of the eight neuronal populations (vertically arranged) of the PD model with fixed in-degrees. Spike times from simulations of the reference network **(A)** and of networks with naively discretized 1-bin **(B)** and 2-bin weights **(C)**. Population-specific distributions of single-neuron firing rates FR **(D)**, coefficients of variation CV of the interspike intervals **(E)**, and spike-train correlation coefficients CC **(F)** from simulations of the reference network (black), as well as networks with 1-bin (blue) and 2-bin weights (yellow). **(G–I)** Mean (solid curves) and standard deviation (shaded areas) of the Kolmogorov-Smirnov (KS) scores *D*_KS_ obtained from distributions in **(D–F)** across five different network realizations. Red: Comparison of simulation results with discretized ($N_{\text{bins}}=1,\ldots ,2^{24}$) and reference weights (with identical random-number generator seed). Black: Comparison of different random realizations of the reference network.

**Figure 4:**
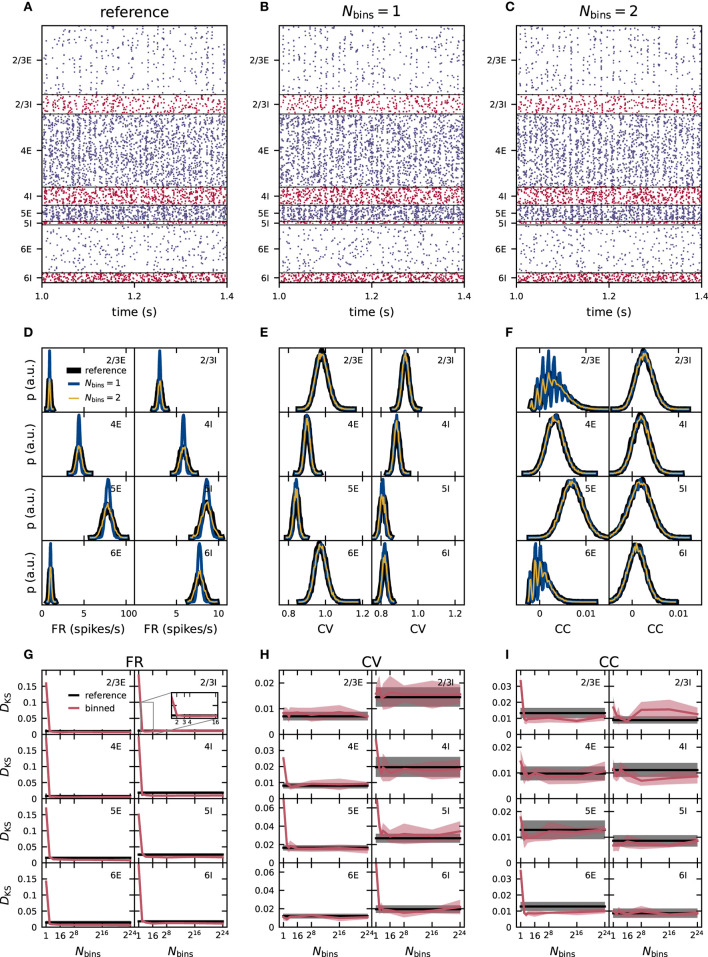
Effect of moment-preserving weight discretization on the spike-train statistics in networks with fixed in-degrees. Same display as in Figure 3.

**Figure 5:**
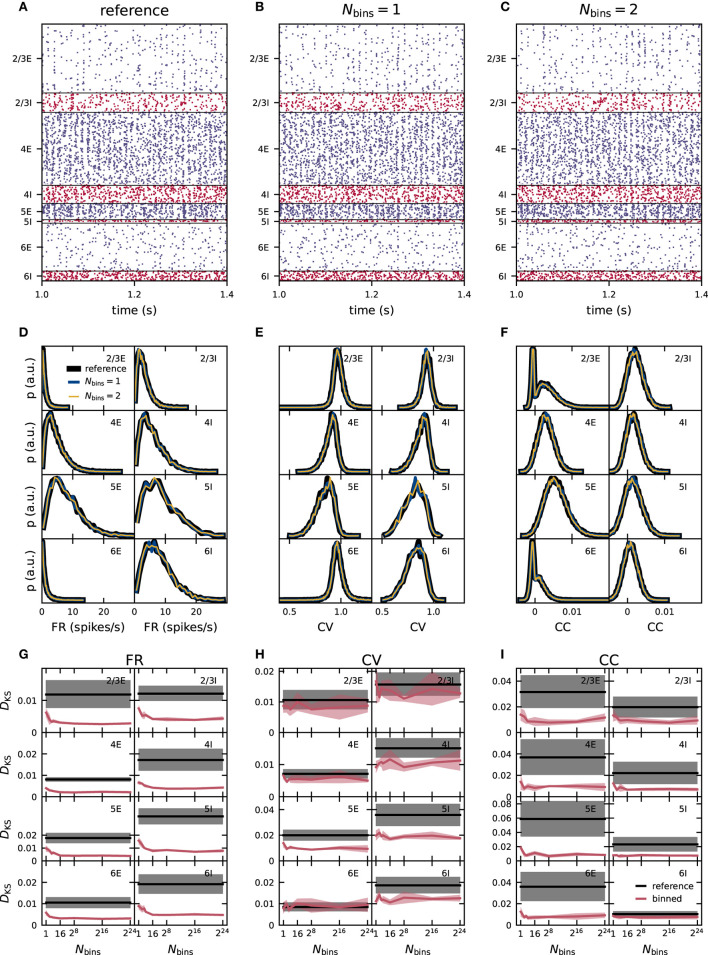
Effect of moment-preserving weight discretization on the spike-train statistics in networks with fixed total numbers of connections. Same display as in Figure 3. Same reference data as in Figure 2.

FR and CV are calculated for all neurons in each neuronal population and the CC for all pairs of 200 distinct neurons in each population. The model validation is based on the distributions of FR, CV, and CC, obtained from these ensembles. The distributions are depicted as histograms with bin sizes 2·(IQR/*n*^1/3^) that are determined using the Freedman-Diaconis rule (Freedman and Diaconis, 1981) based on the inter-quartile range IQR and the sample size *n*. For the histograms depicted in Figures 2–5, the bin size is calculated for the data obtained from the respective reference networks with continuous weight distribution and then used for all shown distributions for one population. In Figure 7, the histogram bin size is obtained from either the longest simulation (60 min; Figures 7A–C), or from the last simulation interval (30–40 min; Figures 7D–F). While visual inspection of the histograms yields a qualitative assessment of the similarity of two distributions *p*(*x*) and *q*(*x*), the Kolmogorov-Smirnov (KS) score

(18) $$\begin{array}{r} D_{\text{KS}}=\sup|P\left(x\right)-Q\left(x\right)| \end{array}$$

provides a quantitative evaluation. The KS score is the maximum vertical distance between the cumulative distribution functions $P\left(x\right)=\int^{x}p\left(y\right)\text{d}y$ and $Q\left(x\right)=\int^{x}q\left(y\right)\text{d}y$ (Gutzen et al., 2018) and thereby is sensitive to differences in both the shapes and the positions of the distributions.

The comparison of the distributions of FR, CV, and CC for a network with continuous weights with those of a network with discretized weights eliminates other sources of variability by using the same instantiation of the random network model. The two networks not only have the same initial conditions, external inputs, connections between identical pairs of neurons, and spike-transmission delays: one by one the weights in the discretized network are the discrete counterparts of the weights in the continuous network (section 2.2).

#### 2.3.2. Observation Duration Determines Specificity of Validation Measures

The criteria that are naturally used to validate a particular model implementation are determined by those features the model seeks to explain. The validation metrics should therefore reflect the specifics of the model, rather than effects that arise from other aspects not directly related to the model under investigation. The example of this study, the model by Potjans and Diesmann (2014), predicts that layer and population specific patterns of FR, spike-train irregularities (ISI CVs), and pairwise correlations are a consequence of the cell-type specific connectivity within local cortical circuits. Distributions of these quantities, therefore, constitute meaningful validation metrics for this model. However, this holds only true if these distributions are obtained such that they primarily reflect the model-specific connectivity, and are not the result of some other trivial effects, for example, those introduced by the measurement process. A standard approach to disentangle such effects is to compare the data generated by the model against those generated by an appropriate null hypothesis where certain model-specific features are purposefully destroyed (see Grün, 2009, for a review of methods for spiking activity and their limitations).

As an example, consider the distributions of spike-train CCs. The PD model predicts that pairwise spike-train correlations are small and distributed around some population-specific non-zero mean, and that these distributions are explained by the specifics of the connectivity. Consider now the alternative hypothesis (null hypothesis) according to which the correlation distributions are fully explained by the distributions of time-averaged FRs and do not reflect any further characteristics of the synaptic connectivity. An instantiation of this null hypothesis is obtained by generating surrogate data from the model data, where the spike times for each neuron are uniformly randomized within the observation interval. Under this null hypothesis, the distributions of FR are fully preserved (Figures 2A,D), but the pairwise correlations on a millisecond timescale (as well as spike-train regularities) are destroyed. For increasing observation time *T*_sim_ → ∞, the distributions of CC approach delta-distributions with zero mean. For finite sample sizes, i.e., finite observation duration *T*_sim_, however, spurious non-zero correlations remain. The correlation distributions obtained under this null hypothesis therefore have some finite width and may be hard to distinguish from the actual model distributions. Indeed, the distributions of spike-train CC obtained from *T*_sim_ = 10 s simulations of the PD model cannot be distinguished from those generated by the null hypothesis introduced above (Figure 2C). Only for sufficiently long observation intervals do the empirical model correlation distributions carry specific information about the network connectivity which is not already contained in the rate distributions (Figure 2F for *T*_sim_ = 15 min).

We conclude that a model validation based on spike-train correlation distributions should be interpreted with care: for short observation duration (e.g., *T*_sim_ = 10 s as used by van Albada et al., 2018, Knight and Nowotny, 2018, Rhodes et al., 2019, and Golosio et al., 2021), any model implementation that preserves the rates but destroys interactions between spike trains would not differ from the reference model with respect to the correlation distributions. Distributions of correlations obtained from short observation periods may however still be useful to rule out that some model implementation erroneously generates correlations that are significantly larger than those generated by the reference model (see e.g., Pauli et al., 2018).

In principle, the same is true for other validation metrics, such as distributions of ISI and their CVs (definitions in section 2.3.1, Figures 2B,E). The surrogate data of this example may suggest all CVs be one, but the finite sample sizes lead to distributions of finite widths and eventually even a shifted mean (as seen in the *T*_sim_ = 10 s case). In the face of finite observation times, one needs to check to what extent these metrics are informative about the specifics of the underlying model, and whether there is actually any chance that some imperfect implementation of the model can lead to deviations from the reference. The comparison with appropriate surrogate data is a straight-forward and established procedure to test this. For our study on weight discretization, this analysis demonstrates that with an observation duration of *T*_sim_ = 15 min, the employed spiking statistics reflect properties of the network model by Potjans and Diesmann (2014).

### 2.4. Software Environment and Simulation Architecture

The simulations in this study are performed on the JURECA supercomputer at the Jülich Research Centre, Germany. JURECA consists of 1,872 compute nodes, each with two Intel Xeon E5-2680 v3 Haswell CPUs running at 2.5 GHz. The processors have 12 cores and support 2 hardware threads per core. Each compute node has at least 128 GB of memory available. The compute nodes are connected via Mellanox EDR InfiniBand.

All neural network simulations in this study are performed using the NEST simulation software (Gewaltig and Diesmann, 2007). NEST uses double precision floating point numbers for the network parameters and the calculations. The simulation kernel is written in C++ but the simulations are defined via the Python interface PyNEST (Eppler et al., 2009). The simulations of the cortical microcircuit are performed with NEST^1^ compiled from the master branch (commit 8adec3c). The compilations are performed with the GNU Compiler Collection (GCC). ParaStationMPI library is used for MPI support. Each simulation runs on a single compute node with 1 MPI process and 24 OpenMP threads.

All analyses are carried out with Python 3.6.8 and the following packages: NumPy (version 1.15.2), SciPy (version 1.2.1), Matplotlib (version 3.0.3), Elephant^2^ (version 0.5.0), and NetworkUnit^3^ (version 0.1.0).

For the source code, see the data availability statement.

## 3. Results

In this study, the evaluation of the role of the synaptic weight resolution is based on the model of a local cortical microcircuit derived by Potjans and Diesmann (2014). The model comprises four cortical layers (L2/3, L4, L5, and L6), each containing an excitatory (E) and an inhibitory (I) neuron population. An 8 × 8 matrix of cell-type and layer specific connection probabilities provides the basis of the connectivity between neurons (Table 5 in Potjans and Diesmann, 2014). Based on this matrix, the present manuscript considers two different probabilistic algorithms to determine which individual neurons in any pair of populations are being connected. First, sections 3.1 and 3.2 use a *fixed in-degree* rule which requires for each neuron of a target population the same number of incoming connections from a source population. Second, in section 3.3, the total number of synapses between two populations is calculated and synapses are established successively until this number is reached. We refer to this latter procedure, which was also employed in the original implementation by Potjans and Diesmann (2014), as the *fixed total number* rule. In both algorithms, synapses are drawn randomly; the exact connectivity realization is hence dependent on the specific sequence of random numbers required for the sampling process, i.e., the choice and the seed of the employed pseudo-random number generators.

In the PD model, a spike of a presynaptic neuron elicits, after a transmission delay, a jump in the synaptic currents of its postsynaptic targets which decay exponentially with time. In the original implementation, the synaptic weights, the amplitudes of these jumps, are drawn from normal distributions when connections are established, and they remain constant for the course of the following state-propagation phase. All excitatory weights are sampled from a normal distribution with the same (positive) mean and the same standard deviation, except for connections from L4E to L2/3E where the mean and standard deviation are doubled. All inhibitory weights are sampled with a different (negative) mean and a different standard deviation.

This study compares the activity statistics obtained from simulations of a reference model with continuous weight distributions with those where the synaptic weights are drawn from the same continuous distributions and subsequently discretized. We refer to an “*N*_bins_ discretization” as the case where the sampled weights are replaced by a finite set of $N_{\text{bins}}\in {\mathbb{N}}^{+}$ discrete values for each of the three weight distributions. As validation measures, we use the time-averaged single-neuron firing rates (FR), the coefficients of variation (CV) of the interspike intervals as a spike-train irregularity measure, and the short-term spike-train correlation coefficients (CC) as a synchrony measure. We quantify the discretization error, i.e., the deviation between the discretized and the reference model, by the Kolmogorov-Smirnov (KS) score *D*_KS_ computed from the empirical distributions of these statistical measures across neurons. To evaluate the significance of the discretization error, we recognize that the model is defined in a probabilistic manner: valid predictions of this model are those features that are exhibited by the ensemble of model realizations. Features that are specific to a single realization are meaningless. Therefore, deviations between realizations of a discretized and the reference model are significant only if they exceed those between different realizations of the reference model. In other words, if the observed KS score between the discretized and the reference model falls into the distribution of KS scores obtained from an ensemble of pairs of reference realizations, the weight discretization does not lead to significant errors with respect to the considered validation measure.

### 3.1. Naive Discretization Distorts Statistics of Spiking Activity

The connectivity of the PD model exhibits different sources of heterogeneity: connections between pairs of neurons result from a random process and distributions govern the creation of their weights and delays. A number of previous studies have shown how such heterogeneities influence neuronal network dynamics (Golomb and Rinzel, 1993; Tsodyks et al., 1993; van Vreeswijk and Sompolinsky, 1998; Neltner et al., 2000; Denker et al., 2004; Roxin, 2011; Roxin et al., 2011; Pfeil et al., 2016). In particular, distributed in-degrees, as implemented with the *fixed total number* rule in the original version of the model by Potjans and Diesmann (2014), can obscure the effects of altered weight distributions which are the primary subject of this study. To isolate the role of the weight distribution, we, therefore, start by investigating a *fixed in-degree* version of the PD model. To assess how discretization of the weights affects the spiking activity in the network, we begin with a simple “naive” discretization scheme: an arbitrary interval is defined around the mean value of the underlying normal distribution (here: ±5 standard deviations) and discretized into a desired number of bins. Each weight sampled from the continuous distribution is replaced by the nearest bin value (for details, see section 2.2).

We use similar measures and procedures as previous studies (e.g., Knight and Nowotny, 2018; van Albada et al., 2018) to compare the activity on a statistical level, but with the major difference here that the network is simulated longer, in fact, 15 min of biological time (see section 3.5). The raster plots in Figures 3A–C show qualitatively similar asynchronous irregular spiking activity in all neuronal populations. The individual spike times, however, are different in the networks with synaptic weights using the reference implementation with double precision in Figure 3A and in the networks with 1- and 2-bin weights in Figures 3B,C, respectively. The dynamics of recurrent neuronal networks similar to the PD model is often chaotic (Sompolinsky et al., 1988; van Vreeswijk and Sompolinsky, 1998; Monteforte and Wolf, 2010). Even tiny perturbations (such as modifications in synaptic weights) can therefore cause large deviations in the microscopic dynamics. Macroscopic characteristics such as distributions of FR, spike-train regularity and synchrony measures, however, should not be affected. Preserving the spiking statistics upon weight discretization is, therefore, an aim of this study.

The distributions of time-averaged FR obtained with 1-bin weights have a similar mean as the reference distribution, but are more narrow in all populations (Figure 3D). In homogeneous networks with non-distributed 1-bin weights, analytical studies predict that all neurons inside one population have the same FR (Brunel, 2000; Helias et al., 2014), in contrast to the reference network with distributed weights and expected rate distribution of finite width. The remaining finite width of the rate distribution obtained from network simulations with 1-bin weights is a finite-size effect and decreases further for larger networks and longer simulation times. For 2-bin weights generated by this naive discretization scheme, the rate distributions are broader than in the reference network (Figure 3D). For several neuronal populations, such as L2/3E or L2/3I, the distributions of the CV of the ISI obtained from networks with discrete weights are similar to those of the reference network (Figure 3E). In other populations, such as L6I, the CV distributions are narrower for 1-bin weights and broader for 2-bin weights, while the mean is preserved. The distributions of CC in the discretized implementations are similar to the reference version for most populations (Figure 3F). Only in L2/3E and L6E, we observe an oscillatory pattern for 1- and 2-bin weights in the region of small correlations. The same oscillatory pattern is also present in the CC distributions of the reference network, but less pronounced (not visible here). As shown in section 2.3.1, this oscillatory pattern is an artifact of the finite observation duration and becomes more eminent in populations with narrow FR distribution with a small mean. The effect is most noticeable in L2/3E and L6E because here the average FR are smallest. In the reference and 2-bin weight networks, the effect is weaker because the rate distributions are broader as compared to the network with 1-bin weight discretization. In the Supplementary Material, we show for the 1-bin case that the CC distributions of surrogate data with randomized spike times exhibit the same oscillatory pattern for the two populations (Supplementary Figure 1).

To quantify the differences in the resulting distributions, we use the KS score. In each case, we compare the distributions of FR, CV, and CC obtained from simulations of networks with binned weights to the reference distributions. To assess the significance of non-zero KS scores, we repeat the comparison analysis for pairs of (random) realizations of the reference network (i.e., different realizations of the connectivity, spike-transmission delays, external inputs, and initial conditions). As simulation results should not qualitatively depend on the specific realization of the probabilistically defined model, all deviations (KS scores) which are of the same size as or smaller than this baseline are insignificant. For all three activity statistics (FR, CV, and CC) the deviations are largest for one and two bins (Figures 3G–I). For around 16 bins, the deviations in all three activity statistics converge toward a non-zero KS score and do not decrease further with any higher number of bins. This residual deviation is the minimal possible deviation for this simulation time. In the *fixed in-degree* network using the naive discretization scheme, these deviations are smaller than the baseline obtained using different network realizations from 16 bins onward. For the kind of network simulation studied here and the specific choice of the binning, 16 bins are therefore sufficient to achieve activity statistics with satisfactory precision. For lower numbers of bins *N*_bins_ ∈ {1, 2, 3, 4}, a pattern appears in almost all populations and for all three statistical measures the deviations from the reference network do not decrease monotonously with an increasing number of bins, but increase from one to two bins, decrease from two to three, and increase again from three to four bins (Figures 3G–I). These differences are highly significant as in several neuronal populations three bins achieve a score value better than the reference obtained using different seeds while four bins do not. The weight discretization procedure (section 2.2) reveals a hint on the origin of this behavior. The naive discretization scheme changes the standard deviation of the weight distributions depending on the number of bins. Three bins achieve a good result by a mere coincidence because due to the choice of the binning interval, the standard deviation of the discrete weights is close to the standard deviation of the reference distributions (see Figure 1). Comparing the KS scores in Figure 3G with the discrepancies between the standard deviation in Figure 1A exhibit the same pattern in both measures.

### 3.2. Moment-Preserving Discretization Preserves Statistics of Spiking Activity

Suspecting that a discrepancy between the standard deviation of the weight distributions in the reference and the binned network results in deviant activity statistics, we derive a discretization method that preserves the standard deviation of the reference weights for any number of bins. This method adapts the width of the interval in which the discrete bins are evenly placed, depending on the number of bins and the reference weight distribution (section 2.2). If the discrepancy in the standard deviation of the weight distributions is indeed the major cause of the errors observed in the activity statistics, the moment-preserving discretization method should substantially reduce these errors. In the 1-bin case, the standard deviation is per definition zero and the optimization procedure cannot be applied. Similarly the optimization procedure is not applied in the cases with 2^16^ and 2^24^ bins because the method becomes more numerically demanding in these cases and no improvement over the naive discretization is expected. Therefore, the shown data for 1, 2^16^, and 2^24^ bins are the same in Figures 3, 4. Already for two bins, the FR, CV, and CC distributions resulting from the moment-preserving discretization visually match the distributions from the reference network in all neuronal populations in Figures 4D–F in contrast to Figures 3D–F. The KS score confirms that the moment-preserving discretization improves the accuracy of simulation with low numbers of bins (Figures 4G–I). A discretization using two bins is sufficient to yield scores of the same order as or even smaller than the baseline resulting from the comparison of different realizations of the reference network. Increasing the number of bins beyond two does not lead to any further improvements for CV and CC. The KS score for the FR decreases slightly (not visible here) up to around 16 bins, from where it remains stationary for all higher number of bins. For the *fixed in-degree* version of the PD model, the accuracy of the simulation is therefore preserved with a 2-bin weight discretization.

### 3.3. Minimal Weight Resolution Depends on In-degree Heterogeneity

So far, we have studied the PD model with fixed in-degrees. In this section, we move on to a model version in which the neuronal populations are connected with the *fixed total number* rule (as originally used by Potjans and Diesmann, 2014), leading to binomial distributions of the numbers of incoming connections per neuron in each population. In comparison to the networks used in sections 3.1 and 3.2, this distribution of in-degrees leads to a heterogeneity across neurons inside one population independent of the weight distributions. We use the moment-preserving discretization scheme and employ the same statistical analysis as in the previous section to determine how this additional network heterogeneity influences the accuracy of network simulations subject to weight discretization.

In Figures 5D–F, the distributions of FR, CV of the ISI and CC using reference weights have different shapes and in most populations increased widths compared to the distributions in the previous *fixed in-degree* network in Figures 4D–F. For all three statistics (FR, CV, and CC) the distributions of the binned network match those of the reference network closely for one and two bins (Figures 5D–F). As before, we quantify the deviations of the simulations with the binned weights from the reference using the KS score (Figures 5G–I). For CV and CC, the score shows no systematic trend with varying number of bins. For the FR, there is a small descend from one to around 16 bins and a stationary score for all higher numbers of bins. Nevertheless, for all three statistical measures, the score values are always smaller or of similar order as the disparity between different realizations of the reference network. Scores computed for the binned and non-binned networks differ only in the synaptic weights and can therefore be smaller than those comparing different realizations of the reference network which have in addition different realizations of the initial conditions, external inputs, connectivity graph, and delays. We conclude that already just one bin successfully preserves the activity statistics in the PD model with distributed in-degrees and using the moment-preserving discretization method this remains true also for higher numbers of bins.

### 3.4. Mean-Field Theory Relates Variability of Weights, In-degrees, and Firing Rates to Minimal Weight Resolution

Two weight bins preserve the activity statistics of the PD model in the *fixed in-degree* network (section 3.2) and one weight bin is sufficient for the network with heterogeneous in-degrees (section 3.3). This observation calls for a deeper look at the influence of weight and in-degree heterogeneity on the firing statistics. In mean-field theory using the diffusion approximation (Fourcaud and Brunel, 2002; Schuecker et al., 2015), the stationary firing response of a neuron *i* with exponential postsynaptic currents is fully determined by the first two cumulants

(19) $$\begin{array}{r} \mu_{i}=\tau_{\text{s}}\underset{j\in X_{i}}{\sum }w_{ij}\nu_{j}, \end{array}$$

(20) $$\begin{array}{r} \sigma_{i}^{2}=\tau_{\text{s}}\underset{j\in X_{i}}{\sum }w_{ij}^{2}\nu_{j}, \end{array}$$

of its total synaptic input current. Here, *X*_*i*_ denotes the population of neurons presynaptic to *i*, ν_*j*_ the stationary FR of presynaptic neuron *j*, *w*_*ij*_ the synaptic weight, and τ_s_ the synaptic time constant. The size of the presynaptic population *X*_*i*_ defines the in-degree *K*_*i*_ = |*X*_*i*_| of neuron *i*. Any heterogeneity in *w*_*ij*_ and *K*_*i*_ (and ν_*j*_) leads to heterogeneous synaptic input statistics μ_*i*_ and $\sigma_{i}^{2}$, and, in turn, to heterogeneous firing statistics. Here, we, therefore, argue that weight discretization preserves the firing statistics across the population as long as it preserves the synaptic-input statistics across the population. Rather than developing a full self-consistent mathematical description of this statistics (van Vreeswijk and Sompolinsky, 1998; Renart et al., 2010; Roxin et al., 2011; Helias et al., 2014), we restrict ourselves to studying the effect of weight discretization on the ensemble statistics of the synaptic-input mean μ_*i*_ and variance $\sigma_{i}^{2}$, under the assumption that the distributions of *w*_*ij*_, *K*_*i*_, and ν_*j*_ are known. For simplicity, we limit this discussion to the first two cumulants of the ensemble distributions, the ensemble mean $\langle x\rangle =\sum_{i\in X}x_{i}/N_{X}$, and variance $\langle \delta x^{2}\rangle =\sum_{i\in X}{\left(x_{i}-\langle x\rangle \right)}^{2}/N_{X}$ of μ_*i*_ and $\sigma_{i}^{2}$ ($x\in \left\{\mu_{i},\sigma_{i}^{2}\right\}$) over all neurons *i* in a population *X* of *N*_*X*_ neurons:

(21) $$\begin{array}{r} \langle \mu \rangle =\tau_{\text{s}}\langle K\rangle \langle w\rangle \langle \nu \rangle , \end{array}$$

(22) $$\begin{array}{r} \langle \sigma^{2}\rangle =\tau_{\text{s}}\langle K\rangle \left({\langle w\rangle }^{2}+\langle \delta w^{2}\rangle \right)\langle \nu \rangle , \end{array}$$

(23) $$\begin{array}{r} \langle \delta \mu^{2}\rangle =\tau_{\text{s}}^{2}[\langle \delta K^{2}\rangle {\langle w\rangle }^{2}{\langle \nu \rangle }^{2}+\langle K\rangle {\langle w\rangle }^{2}\langle \delta \nu^{2}\rangle \\ \;+\langle K\rangle \langle \delta w^{2}\rangle \left({\langle \nu \rangle }^{2}+\langle \delta \nu^{2}\rangle \right)], \end{array}$$

(24) $$\begin{array}{r} \langle \delta {\left(\sigma^{2}\right)}^{2}\rangle =\tau_{\text{s}}^{2}[\left(\langle \delta K^{2}\rangle -\langle K\rangle \right){\left({\langle w\rangle }^{2}+\langle \delta w^{2}\rangle \right)}^{2}{\langle \nu \rangle }^{2}\\ \;+\langle K\rangle \langle w^{4}\rangle \left({\langle \nu \rangle }^{2}+\langle \delta \nu^{2}\rangle \right)]. \end{array}$$

The above expressions rely on Wald's equation^4^ (Wald, 1944), the Blackwell-Girshick equation^4^ (Blackwell and Girshick, 1946), and general variance properties. Note that in previous works on heterogeneous networks, the population variance 〈δ(σ^2^)^2^〉 of the input variance is often neglected (Renart et al., 2010; Roxin et al., 2011; Helias et al., 2014). Roxin et al. (2011) moreover neglect the dependence of 〈σ^2^〉 on the weight variance 〈δ*w*^2^〉. While the ensemble measures in Equations (21)–(24) can be computed for the whole neuronal network, it is more conclusive to use population-specific ensemble measures computed individually for each pair of source *X* and target population *Y*. With this approach, 〈*K*〉 and 〈δ*K*^2^〉 refer to the mean and the variance of the number of inputs from population *X* across all neurons in the target population *Y*, 〈*w*〉 and 〈δ*w*^2^〉 refer to the mean and the variance of the weights of all connections from *X* to *Y*, and 〈ν〉 and 〈δν^2^〉 refer to the mean and the variance of the FR across neurons in the source population *X*. Deriving these population-specific measures is possible because μ and σ^2^ given in Equations (19) and (20), respectively, decompose into the contributions of the different source populations. Besides, we assume that *K*_*i*_ and *w*_*ij*_ are drawn independently from their respective distributions, and the rates ν_*j*_ are also assumed to be independent.

For each of the ensemble measures in Equations (21)–(24), we define a discretization error

(25) $$\begin{array}{r} \varepsilon_{N_{\text{bins}}}\left(x\right)=\frac{\left|x_{\infty }-x_{N_{\text{bins}}}\right|}{\left|x_{N_{\text{bins}}}\right|} \end{array}$$

as the normalized deviation of the measure *x*_*N*_bins__ in a network with *N*_bins_ weight bins from its counterpart *x*_∞_ in the network with the reference weight distribution. In Table 5, we summarize ε_*N*_bins__ for all four ensemble measures to assess deviations introduced by weight discretization to one and two bins according to the moment-preserving scheme. In the 2-bin case, 〈*w*〉 and 〈δ*w*^2^〉 are the same for the reference and binned networks; in the 1-bin case, however, only 〈*w*〉 is preserved while 〈δ*w*^2^〉 vanishes by definition. The term 〈*w*^4^〉 in Equation (24) evaluates for the normal reference weight distribution to 〈*w*〉^4^ + 6〈*w*〉^2^〈δ*w*^2^〉 + 3〈δ*w*^2^〉^2^, for one bin to 〈*w*〉^4^, and for two bins to 〈*w*〉^4^ + 6〈*w*〉^2^〈δ*w*^2^〉 + 〈δ*w*^2^〉^2^. Therefore, the results in the fourth column of Table 5 are only valid for a normal reference weight distribution, while the first three columns are valid independent of the shape of the weight, in-degree, or FR distribution. For simplicity, the rate distributions are here assumed to be similar in the reference and the binned networks. The mean-field theory, in general, relates the fluctuating synaptic input to the distribution of output spike rates by a self-consistency equation such that any change of parameters changes both. Here, we go with the assumption of similarity as we are interested in finding binned networks yielding similar spiking statistics as the reference. Consequently, $\varepsilon_{1}\left(\langle \mu \rangle \right)=\varepsilon_{2}\left(\langle \mu \rangle \right)=\varepsilon_{2}\left(\langle \sigma^{2}\rangle \right)=\varepsilon_{2}\left(\langle \delta \mu^{2}\rangle \right)=0$, since all these measures only depend on quantities that are the same in networks with the reference weight distribution and their binned counterparts. Non-zero table entries result from cases where the respective quantities do not cancel.

**Table 5:** Discretization error of the synaptic-input statistics for 1- and 2-bin discretization.

*x*	〈μ〉	〈σ^2^〉	〈*δμ*^2^〉	〈δ(σ^2^)^2^〉
ε_1_(*x*)	0	*j*	$j\cdot \frac{1+f}{k+f}$	$j\cdot \left(2+j\right)\cdot \left(1+2\frac{1+f}{k+f}\right)$
ε_2_(*x*)	0	0	0	$\frac{2j^{2}}{4j+\frac{k+f}{1+f}{\left(1+j\right)}^{2}}$

*Discretization error ε_N_bins__(x) as defined in Equation (25) for the four ensemble measures Equations (21)–(24) (columns), for networks with N_bins_ = 1 and N_bins_ = 2 weight bins (rows). The parameters j: = 〈δw^2^〉/〈w〉^2^, f: = 〈δν^2^〉/〈ν〉^2^ and k: = 〈δK^2^〉/〈K〉 denote the squared variation coefficients of the synaptic weights and the firing rates, and the Fano factor of the in-degrees, respectively*.

Conventional mean-field theory captures the mean and the variance of the input fluctuations to describe the dynamical state of a recurrent random spiking neuronal network. This is sufficient to predict characteristics of network dynamics like the mean spike rate, the pairwise correlation between neurons, and the power spectrum. Therefore, if the deviations in Table 5 of μ and σ^2^ are small, the activity statistics in the network are expected to be preserved. Right off the bat, the 2-bin discretization seems more promising, because three of the four ensemble averages considered here evaluate to zero by definition. This holds true for any in-degree, weight or FR distribution as long as the 2-bin discretization preserves mean and standard deviation of the weight distribution. In the 1-bin case, the deviation of 〈σ^2^〉 still depends on the spread of synaptic weights without any further additive terms or scaling. In particular, the term does not depend on whether the in-degrees are distributed or not. In networks with a large spread of synaptic weights, a 1-bin weight discretization is therefore always insufficient. The third column of Table 5 considers 〈δμ^2^〉, the variance of the means of the membrane potential across the population. Again, the deviation of this value from the reference evaluates to exactly zero for the 2-bin case. For a single bin, however, a more complex term remains. For small or no variability in the number of incoming synapses, the deviation of 〈δμ^2^〉 in the 1-bin case depends on the width of the weight distribution, but the more the in-degrees are distributed, the smaller this dependence becomes; for a high variability of the in-degree *k* → ∞ with *k*: = 〈δ*K*^2^〉/〈*K*〉 the deviation goes to zero even in the 1-bin case. In that case, the variability of the mean membrane potentials caused by the distributed in-degrees is so large that the variability of the weight distribution does not matter. The deviations of 〈δ(σ^2^)^2^〉, which quantify the variability of the magnitude of the membrane potential fluctuations across the population, are non-zero for both 1- and 2-bin discretization. For one bin, a high in-degree variability *k* → ∞ leads to a residual deviation *j*·(2 + *j*) that only depends on the relative spread *j*: = 〈δ*w*^2^〉/〈*w*〉^2^ of the reference weight distribution. For two bins, the respective deviation declines with an increasing variability of the in-degrees.

**Figure 6:**
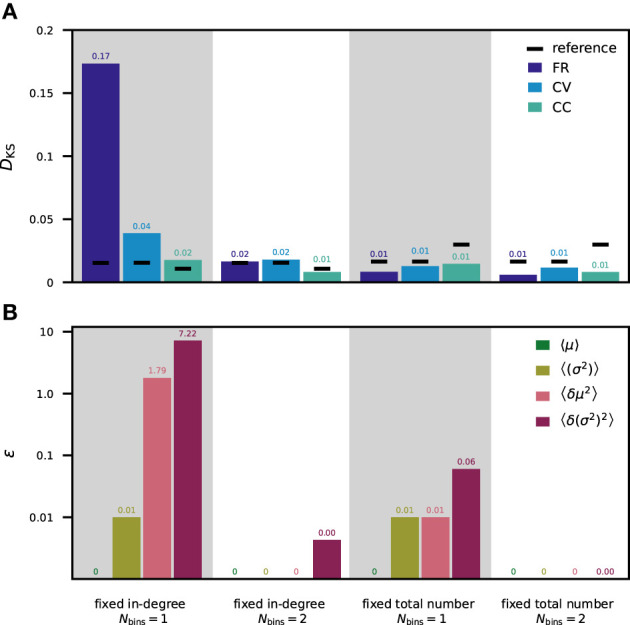
Statistics of spiking activity and discretization errors of the synaptic-input statistics. **(A)** Mean over all neuron populations of the *D*_KS_ scores (for one fixed network realization) calculated as in Figures 4, 5 for FR (dark blue), CV (light blue), and CC (turquoise). Mean reference is shown in black. **(B)** Mean over all pairs of source and target populations of ε values calculated as in Table 5 for the averaged input mean 〈μ〉 (green), averaged input variance 〈σ^2^〉 (olive green), the population variance of the input mean 〈δμ^2^〉 (rose), and the population variance of the input variance 〈δ(σ^2^)^2^〉 (purple). Logarithmic *y*-axis used for ε. First column: *fixed in-degree* network with 1-bin weights. Second column: *fixed in-degree* network with 2-bin weights. Third column: *fixed total number* network with 1-bin weights. Fourth column: *fixed total number* network with 2-bin weights. All ε values vanishing by construction are marked as “0” without decimals, all others are rounded to two decimal places.

For a direct comparison of the theoretical approach and results obtained from analyzing simulated data, we evaluate the terms in Table 5 with parameters and simulation results of our tested network models with moment-preserving weight discretization (Figures 4, 5). The contributions of the firing rates are numerically computed based on measured FR from simulations with the reference weight distribution. Figure 6 is arranged such that the KS scores of the simulated spiking activity in Figure 6A can be directly compared to the computed deviations in the neuron input fluctuations in Figure 6B for the *fixed total number* and *fixed in-degree* networks with one and two weight bins. Both the KS scores and the ε values are here averaged over populations. The ε values for each individual pair of source and target population are shown in the Supplementary Material (Supplementary Figure 2). In the PD model (Potjans and Diesmann, 2014), the standard deviations of the weights are 10% of the mean values, resulting in *j* = 0.01. With *fixed total number* connectivity (multapses allowed), the in-degrees are binomially distributed with a mean of *S*_*YX*_/*N*_*Y*_ and a variance of *S*_*YX*_/*N*_*Y*_(1 − 1/*N*_*Y*_), where *S*_*YX*_ is the total number of synapses between source *X* and target population *Y* and *N*_*Y*_ is the number of neurons in the target population (Senk et al., 2020b). The Fano factor of the in-degrees is, therefore, *k* = 1 − 1/*N*_*Y*_. The *fixed in-degree* scenario simplifies *k* = 0 as 〈δ*K*^2^〉 = 0.

In the *fixed total number* network with one weight bin, the normalized deviations of all of the considered ensemble measures are very small (ε_1_ < 0.1). This is in line with the corresponding KS scores of the spiking activity being all below the reference. In contrast, the *fixed in-degree* network using one weight bin exhibits large discretization errors: values above 1 for 〈δμ^2^〉 and even above 10 for 〈δ(σ^2^)^2^〉 are observed in some populations. These deviations explain the differences in spiking statistics seen in Figure 6A. Using two weight bins, all considered ensemble averages have negligible deviations corroborating the respective observations of negligible deviations in simulation activity statistics.

### 3.5. Observation Duration Determines Validation Performance

**Figure 7:**
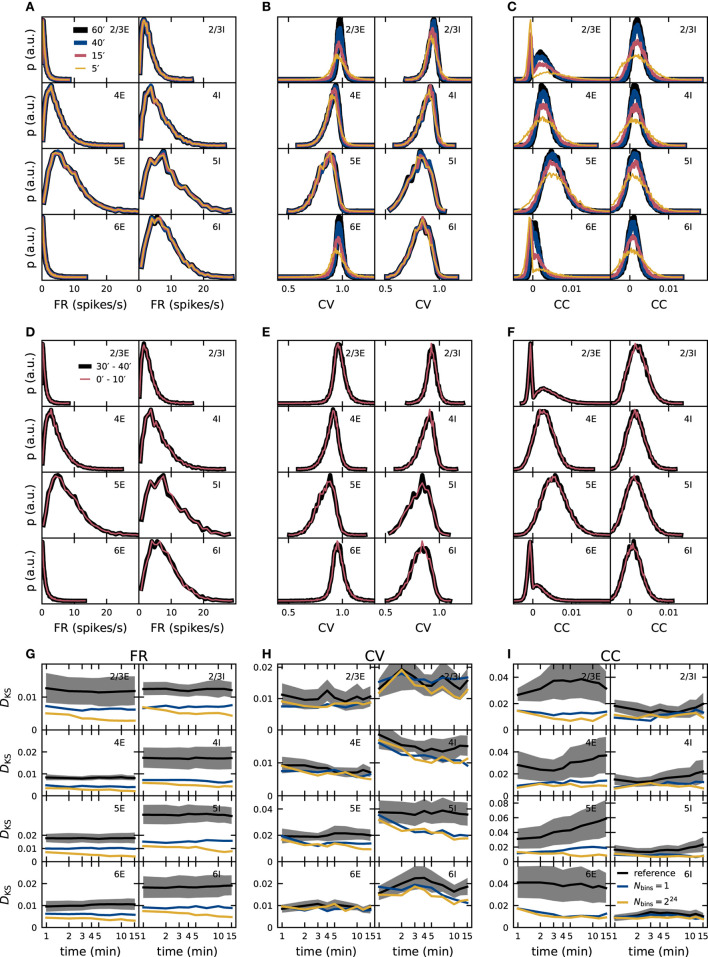
Dependence of validation performance on observation interval and duration. Population specific distributions of single-neuron FR **(A,D)**, CV of the ISI **(B,E)**, and spike-train CC **(C,F)** for different observation durations (5, 15, 40, 60 min; **A–C**) and different observation intervals ([0, 10]min, [30, 40]min) with identical duration **(D–F)**. Dependence of validation performance (KS score *D*_KS_ of distributions obtained from simulations with discretized and double-precision weights) for single-neuron FR **(G)**, CV of the ISI **(H)**, and spike-train CC **(I)** on observation duration *T*_sim_ with 1-bin (blue) and 2^24^-bin weights (yellow). Black traces and gray band represent the mean and standard deviation of KS scores computed with pairs of five random realizations of the reference model.

The dynamical characteristics of any neuronal network model are exposed only if the observation duration is sufficiently long. As shown in section 2.3.2, an observation duration of 15 min is sufficient to ensure that the distributions of spike-train correlation coefficients of the PD model are distinguishable from those obtained for uncorrelated spike trains. So far, however, it remains unclear whether the distributions of the statistical measures are converged, and if our results on the role of quantized synaptic weights are robust with respect to the observation duration. To investigate the convergence behavior of our validation metrics, we analyze simulated data of up to one hour of the model time of the PD model (Figures 7A–C). A completely converged distribution is defined as independent of time when its shape does not change any more if more data is added. FR distributions converge fast; no difference is visible if analyzing only 5 min of the data or the full hour. In contrast, the shape of the distributions of CC still changes after 40 min for all populations and appears not to have converged for the entire data recorded. The behavior of the CV distributions is population-specific: a higher average firing rate leads to more spike data entering the computation of the CVs which results in a faster convergence with simulated model time. The convergence of distributions from low-firing neurons in L2/3E, L2/3I, and L6E, for instance, is slow.

To rule out that the underlying network dynamics change qualitatively over time, which would have been a simple explanation for changes in the distributions, we compare data from two 10 min intervals separated by 20 min: the distributions match for all three metrics (Figures 7D–F). Convergence of the network dynamics to a stationary state happens in fact on a much smaller time scale in the PD model network, and we avoid distortions due to startup transients by always excluding the very first *T*_trans_ = 1 s of each simulation from the data analyzed. To achieve the same interval lengths, also the first second of the [30, 40]min interval is excluded.

These findings make it apparent that only comparisons between simulations of equal model time intervals are meaningful. Choosing a sufficient length for the time intervals such that model specifics are not overshadowed by finite-data effects is a non-trivial task that depends on the network model itself but also on the statistical measures applied, as shown in Figures 2, 7. There are a couple of possible approaches for this endeavor:

1. The conceptually easiest way is to simulate for very long periods of biological times (e.g., more than one hour) until all calculated statistical distributions are converged. Because most complex neuronal network simulations require wall-clock times much longer than the model time simulated on modern HPC systems, this approach is unfeasible until accelerated hardware is available (Jordan et al., 2018).
2. Otherwise one can restrict the analysis to statistics that are less impacted by finite data biases (e.g., the time-averaged FR in Figure 7A). The drawback of this approach is that a thorough validation relies on a number of complementary metrics as decisive model-specific differences may only become evident with some measures and not others (Senk et al., 2017).
3. One can also derive analytical relations for the convergence behavior of certain observables and fit them to a series of differently long simulations. In this way, the true value of the observable can be estimated without finite data biases, as was, e.g., performed in Dahmen et al. (2019).
4. The strategy employed in this study is the following: if qualitative findings are the important parts of the study, then one can first guess a long enough simulation time and perform the study with this. Afterward one has to confirm that the specific measurements to uphold these findings are already converged also for shorter time scales than employed in the study. Figures 7G–I shows the KS scores obtained for a network with *fixed total number* connections but for different simulation durations. Also for shorter simulation times than 15 min the score of a simulation with one bin is below the reference and therefore has acceptable accuracy, while the improvement in accuracy when going to a high number of bins is only small. The qualitative finding that weight discretizations with one bin are sufficient for this network can therefore be upheld also for much shorter simulation times and is unlikely to change for longer times. The drawback of this approach is that one can only confirm in retrospect if the chosen simulation time was sufficient enough, but if one finds the opposite one would have to perform the analysis again for longer simulation times.

## 4. Discussion

This study contributes to the understanding of the effects of discretized synaptic weights on the dynamics of spiking neuronal networks. We found the lowest weight resolution that can maintain the original activity statistics for two derivatives of the cortical microcircuit model of Potjans and Diesmann (2014). In general, the discretization procedure must preserve the moments of the reference weight distributions. In networks where all neurons within one population receive the same number of synaptic inputs, the variability in synaptic weights constitutes the dominant source of input heterogeneity. In this case, the weight discretization has to account for both the mean and the variance of the normal reference weight distributions. In such networks, two discrete weights are sufficient for each pair of populations to preserve the population-level statistics. In networks where the neurons inside the same neuronal population receive different numbers of inputs, the variability in in-degrees may play a major role in the population-level statistics. In the PD model with binomially distributed in-degrees, the in-degree variability is dominating the weight variability such that the original weights can be replaced by their mean value without changing the population-level statistics. The study outlines a mean-field theoretical approach to relate synaptic weight and in-degree heterogeneities to the variability of the synaptic input statistics which, in turn, determines the statistics of the spiking activity. We show that this relationship qualitatively explains the effects of a reduced synaptic weight resolution observed in direct simulations. Finally, the work sheds light on the convergence time of the activity statistics. For a meaningful validation, the simulated model time needs to be long enough such that the statistics are not dominated by effects of finite sample sizes and instead are sufficiently sensitive to distinguish model specifics from random outcomes.

In our approach, synaptic weights are stored with the full floating point resolution the computer hardware supports and all computations are carried out using the full resolution of the floating point unit of the processor. Discretization just refers to the fact that a synaptic weight only assumes one of a small set of predefined values. Thus, for the price of an indirection, only as many bits are required per synapse as needed to uniquely identify the values in the set: one bit for two values, 2 bit for four values. In the cortical microcircuit model of Potjans and Diesmann (2014), the recurrent weights are drawn from one of three distinct distributions (section 2.1). As these weights can be replaced by the respective mean weight without affecting the activity statistics, it is sufficient to store only three distinct weight values (one for each synapse type) rather than the weights for all existing synapses. Based on the 64-bit required for the representation of each weight of the 298, 880, 941 recurrent connections in the cortical microcircuit model with *fixed total number* connectivity, this reduces the memory demand of the network by 2.39 GB (about 15% of the full-resolution reference). This reduction scales linearly with the number of synapses, such that the memory saving potential increases for larger networks. In NEST, this can be achieved by using three synapse models derived from the static_synapse_hom_w class. If several weights are required for each group of neurons, as is the case for *fixed in-degree* connectivity, there exists at present no practical implementation in NEST or neuromorphic hardware that can fully utilize a similar memory saving potential. More research is required on suitable interfaces for the user; the domain specific language NESTML (Plotnikov et al., 2016) offers a perspective.

The synaptic weight resolution can be substantially reduced if the discretization procedure accounts for the statistics of the reference weights. Simulation architectures which allow users to adapt the synaptic weight resolution to the specific network model are therefore preferable to those where the weight representation is fixed. This seems to advocate the use of a mixed precision approach in neuromorphic hardware, in which the synaptic weights are implemented with a lower resolution while the computations are performed with higher numerical precision. An opportunity for future development is to determine which calculation precision is required. While it is possible to achieve comparable network dynamics with 32-bit fixed point arithmetic (van Albada et al., 2018), a minimum bit limit has not been identified, yet.

This study is restricted to non-plastic neuronal networks that fulfill the assumptions underlying mean-field theory as presented, e.g., in Brunel (2000), including heterogeneous networks as studied in Roxin et al. (2011). In such networks, the distribution of synaptic inputs across time can be approximated by a normal distribution (diffusion approximation) such that the statistics of the spiking activity is fully determined by the mean and the variance of this distribution. In general, the approximation becomes more applicable for larger networks or rather larger in-degrees. The PD model has realistic in-degrees (on the order of 10^4^) and we expect that our method is less applicable to a strongly down-scaled version of the model. However, we assume that our main results regarding weight discretization are transferable to other, non-spiking network types which also fulfill the mean-field assumptions, e.g., networks of binary or rate-based neurons; for a mapping between spiking and rate-based neurons see Senk et al. (2020a). Such a transfer requires to reconsider the validation criteria since the CV distribution, for instance, is not defined for rate neurons. The mean-field assumptions rule out spiking networks with low FR, or correlated activity, as well as spiking networks with strong synaptic weights. A number of recent experimental studies revealed long-tailed, non-Gaussian synaptic weight distributions in both hippocampus and neocortex. Here, few individual synapses can be orders of magnitude stronger than the median of the weight distribution (for a review, see Buzsáki and Mizuseki, 2014). Theoretical studies demonstrate that such long-tailed weight distributions can self-organize in the presence of synaptic plasticity (Teramae and Fukai, 2014), and result in distinct dynamics not observed in networks of the type studied here (Teramae et al., 2012; Iyer et al., 2013; Kriener et al., 2014). It remains to be investigated to what extent our conclusions translate to such networks. The study by Teramae and Fukai (2014) indicates that the overall firing statistics in simple recurrent spiking neuronal networks with long-tailed weight distributions can be preserved in the face of a limited synaptic weight resolution, provided this resolution does not fall below 4 bit. Our study employs a uniform discretization of synaptic weights with equidistant bins of identical size. For asymmetric, long-tailed weight distributions, non-uniform discretizations could prove beneficial. In this context, the k-means algorithm may constitute a potential approach (Muller and Indiveri, 2015).

The connectivity of the models considered in this study is fixed and does not change over time. If, on a given hardware architecture, memory is scarce but computations are cheap, the connectivity of such static networks can be implemented using an alternative approach: connectivity data such as weights, delays, and targets do not need to be stored and retrieved many times, but can be procedurally generated for each spike during runtime using a deterministic pseudo-random number generator. In particular, in the case where a single synaptic weight is sufficient to describe the projection between two populations, the effort reduces to the procedural identification of the target neurons. This technique has been applied, for instance, by Eugene M. Izhikevich to simulate a large thalamocortical network model on an HPC cluster^5^, or more recently by Knight and Nowotny (2021) to run a model of vision-related cortical areas (Schmidt et al., 2018) on GPUs, as well as by Heittmann et al. (2020) for a PD model simulation using the IBM Neural Supercomputer (INC-3000) based on FPGAs. Network models with synaptic plasticity, however, require the storage of weights because they are updated frequently during a simulation. Plastic network models are crucial to study slow biological processes such as learning, brain adaptation, and rehabilitation as well as brain development (Morrison et al., 2008; Tetzlaff et al., 2012; Magee and Grienberger, 2020). The present study focuses on the network dynamics at short time scales where plasticity may be negligible. An earlier study already assessed the effect of low weight resolutions in networks with spike-timing dependent plasticity (Pfeil et al., 2012). Further studies need to investigate to what extent a reduced synaptic weight resolution compromises the dynamics and function of plastic neuronal networks. Recent studies indicate that good model performance could be achieved by weight discretization methods based on stochastic roundings (Gupta et al., 2015; Muller and Indiveri, 2015). Stochastic rounding could be implemented in memristive components with probabilistic switching, thus requiring no extra random number generators (Muller and Indiveri, 2015). It would also be interesting to study to what extent discrete weights affect the memory capacity in functional networks (Gerstner and van Hemmen, 1992; Seo et al., 2011). This problem is closely linked to the question of whether weight discretization limits the capabilities of neuronal networks to produce different spatiotemporal activity patterns (Kim and Chow, 2018). The capabilities for discretization in functional networks depend highly on the discretization method (Senn and Fusi, 2005; Gupta et al., 2015; Muller and Indiveri, 2015) and also the neuron models involved. Recently, Cazé and Stimberg (2020) showed that non-linear processing in dendrites enables neurons to perform computations with significantly lower synaptic weight resolution than otherwise possible. Therefore, a principled approach to discretization methods and an adequate selection of performance measures are necessarily dependent on the respective tasks.

A large body of modeling studies treats synaptic weights as continuous quantities that can assume any real number within certain bounds. However, it is known for long that neurotransmission in chemical synapses is quantized—a consequence of the fact that neurotransmitters are released in discrete packages from vesicles in the presynaptic axon terminals. The analysis of spontaneous (miniature) postsynaptic currents, i.e., postsynaptic responses to the neurotransmitter release from single presynaptic vesicles, reveals that the resolution of synaptic weights is indeed finite for chemical synapses. Malkin et al. (2014), for example, show that the amplitudes of spontaneous excitatory postsynaptic currents recorded from different types of excitatory and inhibitory cortical neurons are unimodally distributed with a peak at about 20 pA and a lower bound at about 10 pA. Note that these results have been obtained despite a number of factors that may potentially wash out the discreteness of synaptic transmission, such as variability in vesicles sizes, variability in the position of vesicle fusion zones, quasi-randomness in neurotransmitter diffusion across the synaptic cleft, and variability in postsynaptic receptor densities. For evoked synaptic responses involving neurotransmitter release from many presynaptic vesicles, and for superpositions of inputs from many synapses, the discreteness of synaptic strengths is obscured and unlikely to play a particular role for the dynamics of the neuronal network as a whole. Hence, nature, too, relies to a large extent on discrete network connection strengths. A better understanding of how system-level learning in nature copes with the discrete and probabilistic nature of synapses will guide us toward effective discretization methods for synaptic weights in neuromorphic computers.

To conclude, porting neuronal network models from multi-purpose computing systems to neuromorphic hardware may require adjustments to the original model description for managing hardware constraints like limited available memory. A rigorous validation procedure assesses the effect of potential adjustments and avoids unwanted behavior. This study makes use of common tools from computational neuroscience including network simulation, statistical data analysis, and a mean-field approach to challenge relevant performance measures of a model under the assumption of a limited synaptic weight resolution, and proposes a strategy for weight discretization without compromising the dynamics. Future work needs to investigate to what extent more complex networks are affected by limiting the weight resolution. In particular, it remains an open question whether synaptic or cell-intrinsic plasticity mechanisms can compensate for this.

## Data Availability Statement

The code to reproduce all figures of this paper is available at Zenodo (https://doi.org/10.5281/zenodo.4696168), further inquiries can be directed to the corresponding author/s.

## Author Contributions

SD performed the simulations and analyzed and visualized the data. SD and JS developed the mean-field theoretical approach. SD was supervised by JS and MD. All authors jointly did the conceptual work, wrote the paper, reviewed the manuscript, and approved it for publication.

## Funding

This project has received funding from the European Union's Horizon 2020 Framework Programme for Research and Innovation under Specific Grant Agreement No. 785907 (Human Brain Project SGA2) and No. 945539 (Human Brain Project SGA3), and the Helmholtz Association Initiative and Networking Fund under project number SO-092 (Advanced Computing Architectures, ACA).

## Conflict of Interest

The authors declare that the research was conducted in the absence of any commercial or financial relationships that could be construed as a potential conflict of interest.

## Publisher's Note

All claims expressed in this article are solely those of the authors and do not necessarily represent those of their affiliated organizations, or those of the publisher, the editors and the reviewers. Any product that may be evaluated in this article, or claim that may be made by its manufacturer, is not guaranteed or endorsed by the publisher.

## Footnotes

^1^https://github.com/nest/nest-simulator.

^2^https://python-elephant.org.

^3^https://github.com/INM-6/NetworkUnit.

^4^Let (*X*_*n*_)_*n*∈ℕ_ be a sequence of real-valued, independent and identically distributed random variables and let *N* be a non-negative integer-value random variable that is independent of the sequence (*X*_*n*_)_*n*∈ℕ_. Suppose that *N* and the *X*_*n*_ have finite expectations. Wald's equation: $\langle \overset{N}{\underset{n=1}{\sum }}X_{n}\rangle =\langle N\rangle \langle X_{1}\rangle$. Blackwell-Girshick equation: $\langle \delta {\left(\langle \overset{N}{\underset{n=1}{\sum }}X_{n}\rangle \right)}^{2}\rangle =\langle \delta N^{2}\rangle {\langle X_{1}\rangle }^{2}+\langle N\rangle \langle \delta {X_{1}}^{2}\rangle$.

^5^https://www.izhikevich.org/human_brain_simulation/why.htm.
