# Supplementary material for: Dynamical Characteristics of Recurrent Neuronal Networks Are Robust Against Low Synaptic Weight Resolution
Source: Front Neurosci. 2021 Dec 24;15:757790. doi: 10.3389/fnins.2021.757790 (PMC8740282; doi:10.3389/fnins.2021.757790)
Supplement: Supplementary file 1 [file Data_Sheet_1.PDF]

frontiers

Supplementary Material

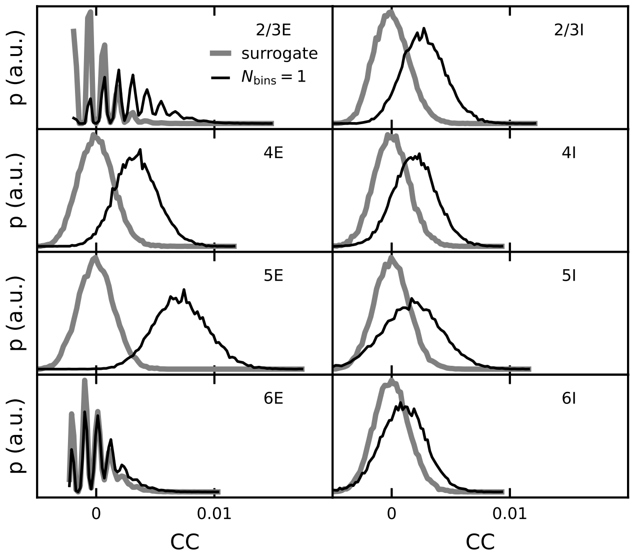

Figure S1: **Finite data reveals discreteness of correlation coefficients.** Distributions of population-specific spike-train correlation coefficients CC. Black: *fixed in-degree* network model with 1-bin weight discretization (same data as in Figures 3 and 4). Gray: surrogate data with randomized spike times, where the total number of spikes of each neuron during the observation duration $T_{\mathrm{sim}} = 15\,\mathrm{min}$ is identical to the former. Oscillatory pattern visible for populations with low firing rate (L2/3E and L6E) is explained in Section 2.3.1.

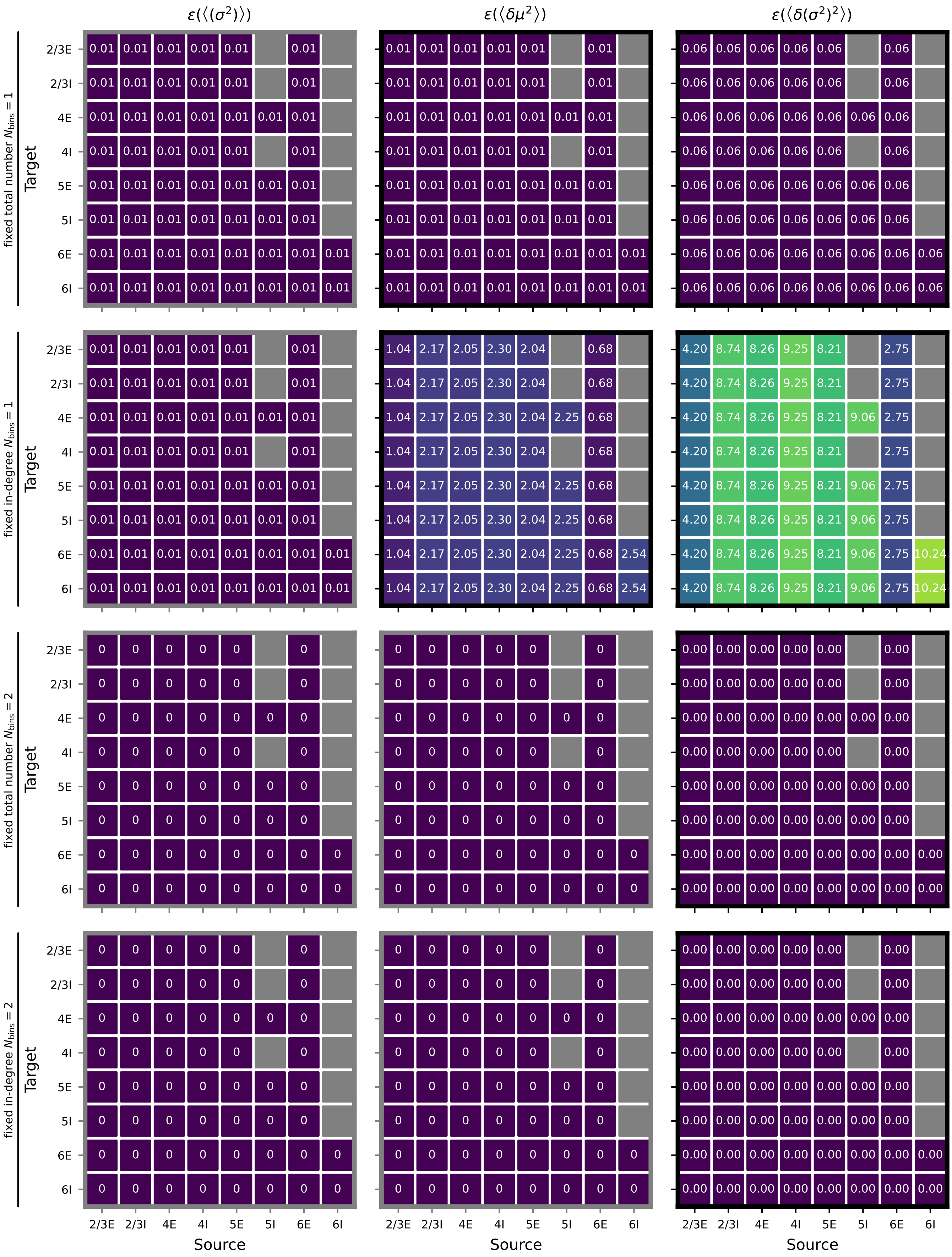
ε(⟨(σ²)⟩)
ε(⟨δμ²⟩)
ε(⟨δ(σ²)²⟩)
fixed total number N_bins = 1
fixed in-degree N_bins = 1
fixed total number N_bins = 2
fixed in-degree N_bins = 2
Target
2/3E 2/3I 4E 4I 5E 5I 6E 6I
Source

Figure S2 *(preceding page)*: **Discretization errors of the synaptic-input statistics in mean-field approximation**. Discretization errors ε of the population-averaged input variance $\left\langle \sigma^2 \right\rangle$ (left column), the population variance of the input mean $\left\langle \delta\mu^2 \right\rangle$ (middle column), and the population variance of the input variance $\left\langle \delta \left(\sigma^2\right)^2 \right\rangle$ (right column) for all pairs of source and target populations. Values are calculated according to Table 5 from weight and connectivity parameters, and from empirical firing-rate distributions obtained in network simulations. First row: *fixed total number* network with 1-bin weights. Second row: *fixed in-degree* network with 1-bin weights. Third row: *fixed total number* network with 2-bin weights. Fourth row: *fixed in-degree* network with 2-bin weights. The discretization error of the population-averaged input mean $\langle \mu \rangle$ vanishes by construction, and is therefore not shown here. Other discretization errors vanishing by construction are marked by “0”. All other errors are rounded to two decimal places. Gray matrix elements indicate unconnected pairs of populations. Discretization errors not depending on the firing-rate distributions are marked with gray frames.
